# RNF112 Facilitates Ubiquitin‐Mediated Degradation of c‐Myc, Suppressing Proliferation, Migration and Lipid Synthesis in Bladder Cancer

**DOI:** 10.1002/advs.202408311

**Published:** 2025-04-03

**Authors:** Kangping Xiong, Siming Chen, Huimin Xu, Sheng Tu, Hong Weng, Yejinpeng Wang, Mingxing Li, Jingtian Yu, Kaiyu Qian, Lingao Ju, Yi Zhang, Yu Xiao, Xinghuan Wang, Gang Wang

**Affiliations:** ^1^ Department of Urology Hubei Key Laboratory of Urological Diseases Zhongnan Hospital of Wuhan University Wuhan 430071 China; ^2^ Department of Obstetrics and Gynecology Ultrasound Zhongnan Hospital of Wuhan University Wuhan 430071 China; ^3^ Center for Evidence‐Based and Translational Medicine Zhongnan Hospital of Wuhan University Wuhan 430071 China; ^4^ Department of Urology Sir Run Run Shaw Hospital of Zhejiang University Hangzhou 310016 China; ^5^ Department of Biological Repositories Human Genetic Resource Preservation Center of Hubei Province Zhongnan Hospital of Wuhan University Wuhan 430071 China; ^6^ Euler Technology ZGC Life Sciences Park Beijing 102206 China; ^7^ Center for Quantitative Biology School of Life Sciences Peking University Beijing 100091 China; ^8^ Wuhan Research Center for Infectious Diseases and Cancer Chinese Academy of Medical Sciences Wuhan 430071 China; ^9^ Medical Research Institute Frontier Science Center of Immunology and Metabolism Wuhan University Wuhan 430071 China

**Keywords:** Bladder cancer, c‐Myc, lipid synthesis, RING finger (RNF) protein 112, ubiquitination

## Abstract

The E3 ubiquitin ligase RNF112 is significantly downregulated in bladder cancer (BLCA) and is correlated with disease progression. In vitro and in vivo studies indicated that RNF112 suppresses BLCA cell proliferation, migration, and lipid synthesis. Mechanistically, RNF112 directly interacts with the MB II domain of MYC through its N‐terminal zinc finger motif, and its catalytic site C97 facilitates K48‐linked polyubiquitination of the K389 residue on the c‐Myc protein, accelerating its degradation. Additionally, this research validated the interaction of c‐Myc with the promoter of ATP citrate lyase (*ACLY*), a central enzyme of lipid metabolism, promoting its transcriptional activity. The restoration of c‐Myc or ACLY expression attenuated the inhibitory effects of RNF112 on BLCA cell growth, migration, and lipid synthesis. In conclusion, this study confirmed that RNF112 suppressed the proliferation, migration, and lipid synthesis of BLCA cells by facilitating the ubiquitin‐mediated degradation of c‐Myc.

## Graphical abstract

RNF112, an E3 ubiquitin ligase, is markedly reduced in bladder cancer (BLCA). It curbs BLCA cell proliferation, migration, and lipid production primarily by enhancing ubiquitin‐dependent degradation of c‐Myc. This degradation inhibits c‐Myc's role in upregulating *ACLY*, a crucial enzyme for lipid synthesis, highlighting RNF112's potential as a therapeutic target in BLCA management.

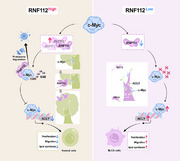

## 1 Introduction

The incidence of bladder cancer (BLCA) exceeds 500 000 cases annually and is predicted to double by 2040, according to the World Health Organization.^[^ ^1^ ^]^ Notably, one‐quarter of newly diagnosed BLCA patients have muscle‐invasive BLCA or metastatic BLCA, which severely impacts patient survival.^[^ ^2^ ^]^ The 5‐year mortality rate for muscle‐invasive BLCA patients with lymph node metastasis exceeds 77%.^[^ ^3^ ^]^ Therefore, exploring new mechanisms of BLCA tumorigenesis and metastasis and developing potential targets for treatment are important for improving the prognosis of patients with BLCA.

The ubiquitin‐proteasome system is critical for tumors and influences the protein stability of several key molecules associated with tumorigenesis, progression, metastasis, and chemotherapy resistance.^[^ ^4^ ^]^ Our previous studies confirmed several E3 ligases that are strongly associated with BLCA proliferation and metastasis.^[^ ^5^ ^]^ The RING finger (RNF) protein family comprises important E3 ligases. Our previous study revealed that RNF126 can facilitate the progression of BLCA by targeting the pivotal protein PTEN for degradation.^[^ ^5b^ ^]^ Subsequent investigations revealed that the expression of RNF112, another member of the RNF protein family, was notably downregulated in BLCA tissues. RNF112 has been reported to interact with several proteins, such as TAR DNA‐binding protein 43 (TDP‐43),^[^ ^6^ ^]^ myeloid leukemia zinc finger (Plzf),^[^ ^7^ ^]^ forkhead box protein M1 (FOXM1),^[^ ^8^ ^]^ and specificity protein 1 (Sp1),^[^ ^9^ ^]^ and regulate their expression or localization. Moreover, the expression and function of RNF112 vary among different tissues. Studies have demonstrated a significant downregulation of RNF112 in malignant glioblastoma multiforme, with low *RNF112* mRNA levels strongly correlated with a negative prognosis. The inhibitory effects of RNF112 on the progression of malignant glioblastoma multiforme are attributed to its influence on p53‐mediated cell cycle regulation.^[^ ^10^ ^]^ In gastric cancer, RNF112 interacts with FOXM1 through its N‐terminal structural domain and promotes its ubiquitination‐mediated degradation, ultimately inhibiting tumor growth and metastasis.^[^ ^8^ ^]^ Nevertheless, there have been no reports on the expression and role of RNF112 in BLCA thus far.

c‐Myc, a transcription factor, is essential for various biological processes, including cell cycle regulation, metabolic reprogramming, epithelial‐mesenchymal transition (EMT), and signal transduction.^[^ ^11^ ^]^ Our previous studies revealed that several key molecules (e.g., POLD1, USP43 and TRAIP) interact with c‐Myc to regulate its ubiquitination and protein stability, thereby affecting BLCA progression.^[^ ^5^, ^12^ ^]^ Lipid metabolism is closely related to the risk, progression, and metastasis of BLCA.^[^ ^13^ ^]^ c‐Myc is a regulator of various lipid metabolism‐related enzymes,^[^ ^14^ ^]^ and whether it is involved in the regulation of lipid metabolism in BLCA and the possible underlying mechanisms remain to be further studied.

This study revealed that RNF112 expression was significantly reduced in BLCA. Overexpression of RNF112 inhibited the proliferation, metastasis, and lipid synthesis of BLCA cells. RNF112 promotes the K48‐linked polyubiquitination of c‐Myc at K389, influencing lipid synthesis in BLCA cells by regulating ATP citrate lyase (*ACLY*) transcription through c‐Myc. These findings suggest that targeting the RNF112/c‐Myc/ACLY axis could be a promising therapeutic strategy for BLCA.

## 2 Results

### 2.1 RNF112 was Downregulated in BLCA

**Figure 1:**
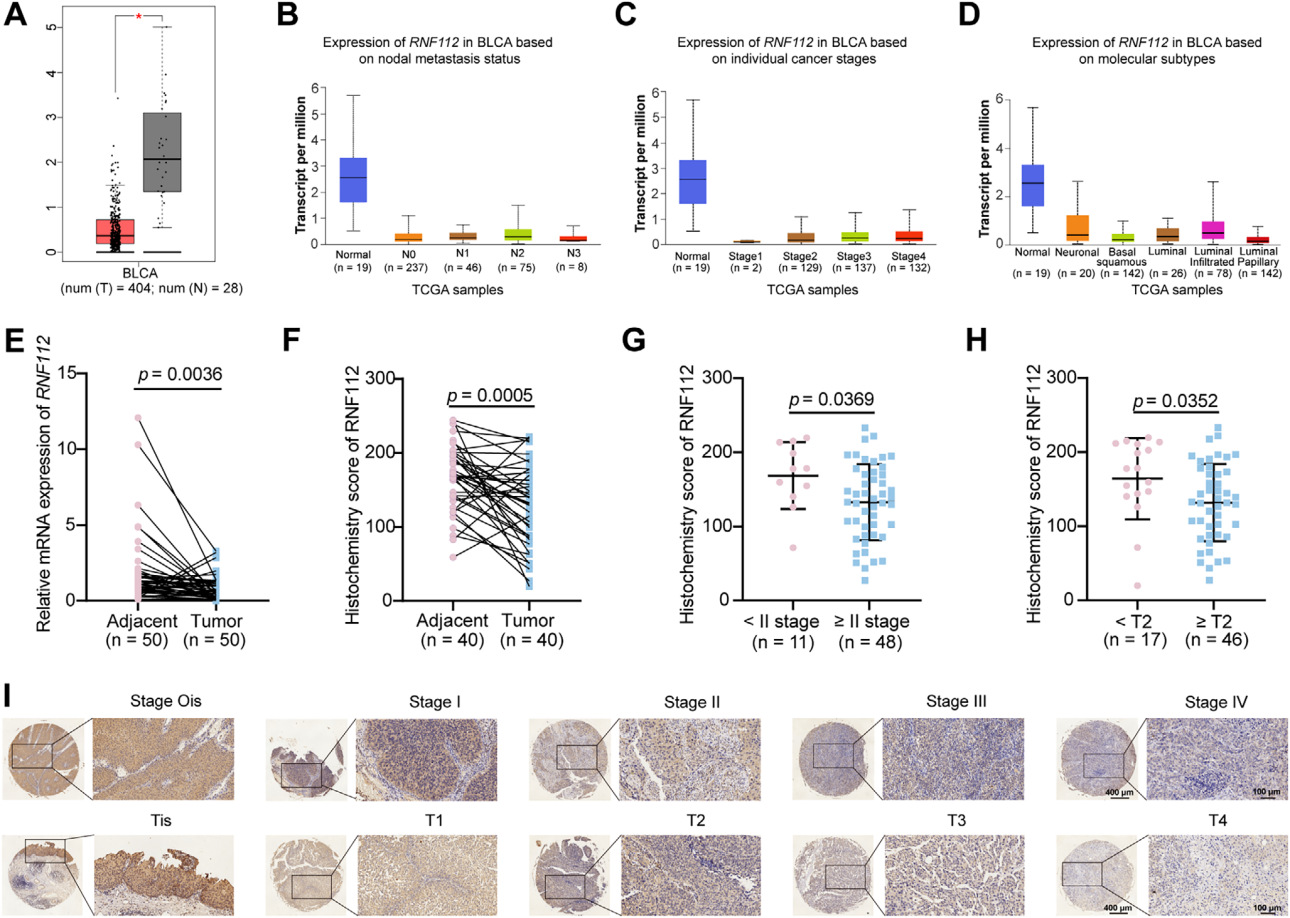
The downregulation of RNF112 expression was negatively associated with the progression of BLCA patients. A) *RNF112* mRNA expression levels in BLCA and normal tissues in the GEPIA dataset^[^ ^12b^ ^]^ (containing the TCGA and GTEx datasets). B–D) *RNF112* mRNA expression levels in BLCA patients according to nodal metastasis status (B), individual cancer stages (C), and molecular subtypes (D) in the UALCAN dataset (containing TCGA datasets). E) *RNF112* mRNA expression levels in BLCA and normal tissues from BLCA patients at Zhongnan Hospital (*n* = 50). F) The histochemistry score was determined by a combination of Aipathwell software and a pathologist. The histochemistry scores of RNF112 in BLCA (*n* = 40) and normal tissues (*n* = 40) in the HBlaU108Su01 cohort were calculated. G,H) Statistical graph of the histochemistry score of RNF112 according to different AJCC stages (H) and T stages (I) in the HBlaU108Su01 cohort. I) Representative IHC images at different AJCC stages and T stages in the HBlaU108Su01 cohort. Scale bar = 400 µm (left); scale bar of enlarged images = 100 µm (right). *p*‐values were determined via paired Student's *t*‐tests (E and F) and two‐tailed unpaired Student's *t*‐tests (G and H). *p < 0.05.

The TIMER2.0 and GEPIA datasets revealed that *RNF112* mRNA was significantly downregulated in most cancers, including BLCA (**Figure** **1**A; Figure S1A, Supporting Information). According to the UALCAN dataset, *RNF112* mRNA expression was downregulated in BLCA tissues with different lymph node metastasis statuses, tumor stages, and molecular subtypes compared with normal tissues (Figure 1B–D).

Moreover, 50 paired BLCA and adjacent tissues collected by our group further confirmed that, compared with that in adjacent tissues, the mRNA expression of *RNF112* was significantly downregulated in BLCA tissues (Figure 1E). Analysis of the cBioPortal database revealed that mutations and deep deletions of *RNF112* are prevalent in BLCA (Figure S1B, Supporting Information), which may account for its low expression. Immunohistochemical (IHC) staining of BLCA tissue microarrays confirmed that the RNF112 protein level was lower in BLCA tissues than in adjacent tissues (Figure 1F; Figure S1C,D, Supporting Information). On the basis of the AJCC or T staging classifications, we categorized patients with BLCA on tissue microarrays into early (Ois‐I) and late (II‐IV) stages or nonmuscle‐invasive bladder cancer (NMIBC) (Tis‐T1) and muscle‐invasive bladder cancer (MIBC) (T2‐T4) stages, respectively. Our results revealed that RNF112 protein levels were significantly lower in late‐stage tissues or MIBC tissues than in early‐stage tissues or NMIBC tissues (Figure 1G–I; and Table S1, Supporting Information). The above results revealed a significant decrease in RNF112 in BLCA, suggesting that RNF112 may play a potential role in BLCA progression.

### 2.2 RNF112 Inhibits BLCA Growth and Metastasis In Vitro and In Vivo

We analyzed the protein expression of RNF112 in a normal human bladder epithelial cell line (SV‐HUC‐1) and various BLCA cell lines (UM‐UC‐3, T24, RT4, SCaBER, 5637, and J82). The expression of RNF112 was lower in all BLCA cell lines than in SV‐HUC‐1 cells. In addition, among the BLCA cell lines, T24 cells presented the lowest expression, whereas 5637 cells presented the highest expression (Figure S2A, Supporting Information). Therefore, we selected T24 and 5637 cells for subsequent experiments.

**Figure 2:**
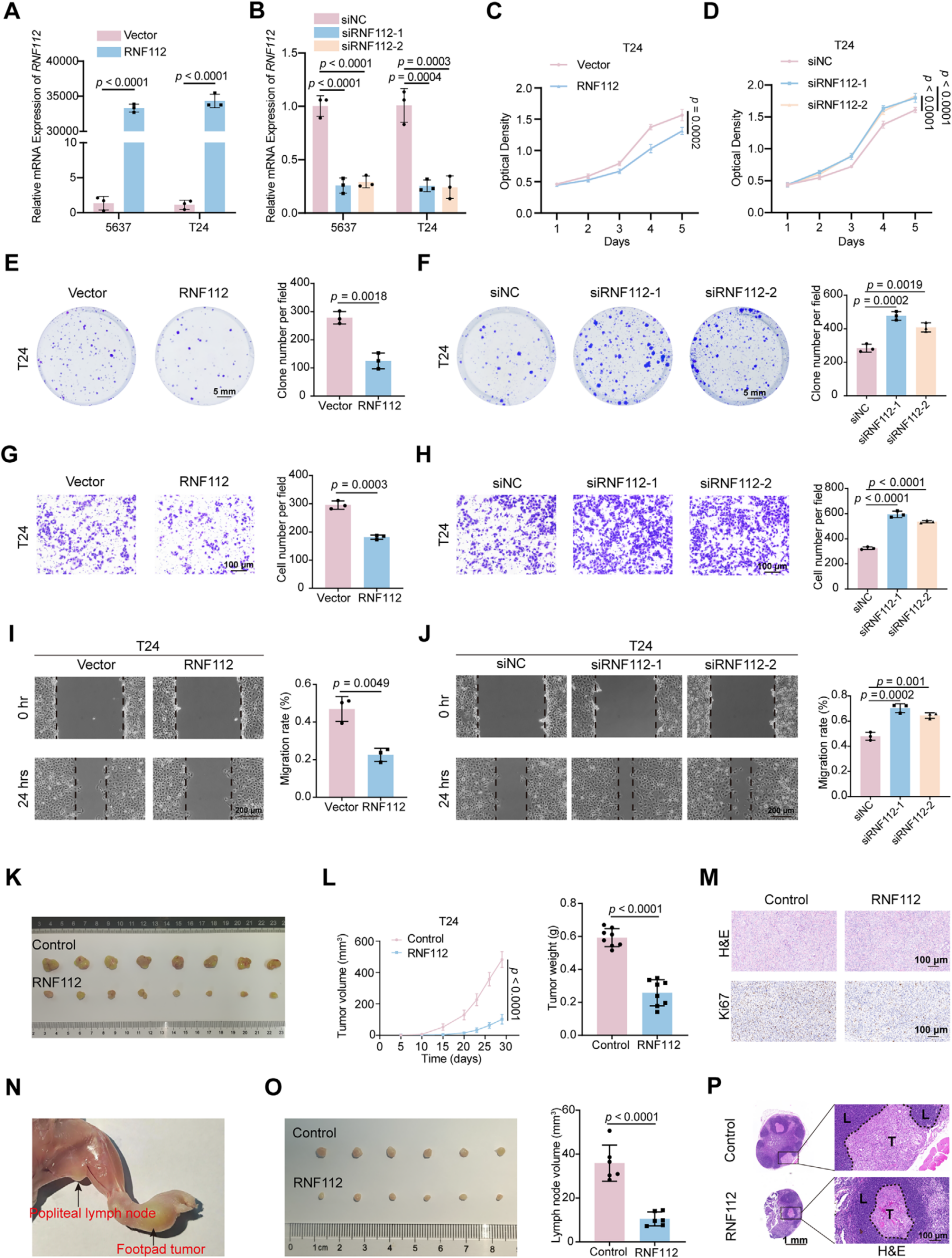
RNF112 inhibits BLCA growth and metastasis in vitro and in vivo. A,B) qRT‐PCR was used to validate the overexpression (A) and knockdown (B) of RNF112 in T24 and 5637 cells (*n* = 3). C,D) The viability of T24 cells with RNF112 overexpression (C) or knockdown (D) was determined by CCK‐8 assays (*n* = 6). E,F) Representative images of plate colony formation assays after the overexpression E) or knockdown (F) of RNF112 in T24 cells and the corresponding statistical graphs (*n* = 3). Scale bar = 5 mm. G,H) Representative images of transwell migration assays after the overexpression (G) or knockdown (H) of RNF112 in T24 cells and the corresponding statistical graphs (*n* = 3). Scale bar = 100 µm. I,J) Representative images of wound healing assays after the overexpression (I) or knockdown (J) of RNF112 in T24 cells and the corresponding statistical graphs (*n* = 3). Scale bar = 200 µm. K) Gross image of subcutaneous tumors in the control and stable RNF112‐overexpressing groups generated from T24 cells (*n* = 8). L) Statistical graphs of the volume (left) and weight (right) of subcutaneous tumors in the control and RNF112 stable overexpression groups. M) Representative images of H&E staining and Ki67 staining of subcutaneous tumors in the control and stable RNF112‐overexpressing groups. Scale bar = 100 µm. N) Representative image of the footpad‐popliteal lymph node metastasis model. O) Gross image of popliteal lymph nodes (left) and corresponding volume statistical graph (right) in the control and RNF112 stable overexpression groups constructed from T24 cells (*n* = 6). P) Representative images of H&E‐stained popliteal lymph nodes in the control and RNF112 stable overexpression groups. L = lymphoid tissue; T = metastatic tumor. Scale bar = 1 mm (left), scale bar of enlarged images = 100 µm (right). *p*‐values were determined via two‐tailed unpaired Student's *t*‐tests (A, C, E, G, I, L, and O) and one‐way ANOVA with Dunnett's multiple comparisons (B, D, F, H, and J).

RNF112 knockdown and overexpression cell models were constructed by transfecting 5637 and T24 cells with siRNA and RNF112 overexpression plasmids, respectively (**Figure** **2**A,B). CCK8 cell proliferation and colony formation assays confirmed that the proliferative ability of BLCA cells was significantly inhibited after overexpression of RNF112, and conversely, knockdown of RNF112 promoted the proliferation of BLCA cells (Figure 2C–F; Figure S2B–E, Supporting Information). Moreover, wound healing assays and transwell migration assays were used to investigate the effect of RNF112 on the metastatic ability of BLCA cells, and the results revealed that overexpression of RNF112 inhibited the metastasis of BLCA cells, whereas knockdown of RNF112 promoted metastasis (Figure 2G–J; Figure S2F–I, Supporting Information). On the basis of our research group's prior experience^[^ ^12a^ ^]^ and previous studies,^[^ ^15^ ^]^ we subsequently performed Western blot analyses to detect changes in the expression of several representative proteins associated with tumor proliferation (Cyclin E1, Cyclin D1, and PCNA) and metastasis (Vimentin, Snail, N‐cadherin, and E‐cadherin) (Figure S2J,K, Supporting Information), which further corroborated the effect of RNF112 on the proliferation and metastasis of BLCA cells.

To validate the above findings in vivo, we constructed a T24 cell line stably overexpressing RNF112 (Figure S3A, Supporting Information). A subcutaneous xenograft model and a footpad popliteal lymph node metastasis model were used to explore the effects of RNF112 on BLCA growth and metastasis in vivo. Subcutaneous xenograft experiments demonstrated that RNF112 reduced the growth rate of BLCA cells in vivo (Figure 2K,L). H&E staining and Ki67 staining of subcutaneous tumor tissues revealed that RNF112 inhibited the proliferative capacity of BLCA cells (Figure 2M). In addition, in the footpad‐popliteal lymph node metastasis model, the RNF112‐overexpressing group had fewer lymph nodes and fewer tumor metastases, suggesting that RNF112 inhibits lymph node metastasis in BLCA in vivo (Figure 2N–P).

### 2.3 RNF112 Inhibits the MYC Pathway and Lipid Synthesis in BLCA

**Figure 3:**
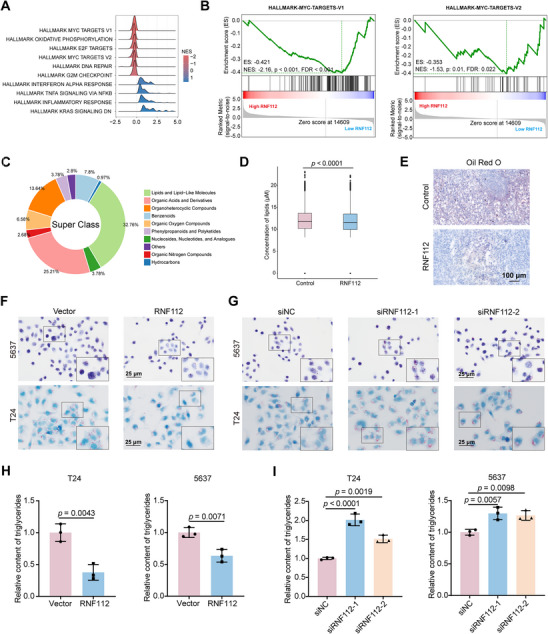
RNF112 inhibits the MYC pathway and lipid synthesis in BLCA. A) Enrichment analysis of the gene expression data obtained from RNA‐seq was performed via hallmark gene sets. The top ten gene sets were selected and sorted from smallest to largest in terms of the normalized enrichment score (NES). B) GSEA of RNA sequencing data showing a negative correlation between RNF112 and MYC‐target V1, as well as between RNF112 and MYC‐target V2. C) Classification and percentage statistics of significantly different metabolites between control and RNF112‐overexpressing T24 cells. D) RNF112 overexpression decreases the total abundance of lipid‐related metabolites. E) Representative images of Oil Red O‐stained tumor tissue from subcutaneous xenografts in the control and RNF112‐overexpressing groups. Scale bar = 100 µm. F,G) Representative images of Oil Red O staining after overexpression (F) and knockdown (G) of RNF112 in T24 and 5637 cells. Scale bar = 25 µm. H,I) Statistical graphs showing the relative triglyceride content after the overexpression (H) or knockdown (I) of RNF112 in T24 and 5637 cells. *p*‐values were determined by two‐tailed unpaired Student's *t*‐test (H) and one‐way ANOVA with Dunnett's multiple comparisons (I).

RNA sequencing was used to explore the potential mechanisms underlying the effects of RNF112 on BLCA. GSEA of hallmark gene sets revealed that several signaling pathways were enriched; therefore, we ranked the enriched pathways according to the normalized enrichment score (NES) value and found that MYC‐target V1 was the top‐ranked pathway (**Figure** **3**A; and Dataset S1, Supporting Information), thus drawing our attention. Moreover, RNF112 was significantly negatively correlated with the MYC‐target V1 pathway (Figure 3B; Figure S3B, Supporting Information). KEGG pathway classification analysis revealed that RNF112 was associated with pathways involved in tumors, lipid metabolism, and signal transduction (Figure S3C, Supporting Information).

Given that RNF112, as an E3 enzyme, has the potential to influence signal transduction by targeting the degradation of key downstream substrates, we focused on the effects of RNF112 on BLCA lipid metabolism. Therefore, we performed an untargeted liquid chromatography‐mass spectrometry (LC‐MS)‐based metabolomics analysis using BLCA T24 cells overexpressing RNF112 and control cells (Dataset S2, Supporting Information). The results revealed that lipids constituted the largest proportion of all differential metabolites (Figure 3C). Moreover, the total abundance of various lipid classes was significantly lower in the RNF112‐overexpressing group than in the control group (Figure 3D). Oil Red O staining revealed that xenograft tumor tissues in the RNF112‐overexpressing group presented lower levels of lipid droplets than did those in the control group (Figure 3E). We also found that the overexpression of RNF112 in 5637 and T24 cells led to a significant reduction in the number of intracellular lipid droplets (Figure 3F), whereas the knockdown of RNF112 caused their accumulation (Figure 3G). Additionally, the overexpression of RNF112 markedly decreased the intracellular triglyceride content (Figure 3H), whereas the knockdown of RNF112 resulted in a significant increase in the level of intracellular triglycerides (Figure 3I). In summary, our results suggest that RNF112 is negatively correlated with the MYC‐targets V1 pathway in BLCA and, in addition, RNF112 significantly inhibits lipid synthesis in BLCA.

### 2.4 RNF112 Interacts with c‐Myc and Promotes its Degradation

Since the effect of RNF112 on the MYC‐target V1 pathway could result from alterations in either the transcriptional levels or protein levels of c‐Myc, we examined the changes in MYC at the mRNA and protein levels in RNF112‐knockdown or RNF112‐overexpressing T24 and 5637 cells. The results indicated that RNF112 did not affect the mRNA level of *MYC* in BLCA cells (Figure S4A,B, Supporting Information). However, RNF112 knockdown increased the protein level of c‐Myc, whereas RNF112 overexpression significantly reduced the protein level of c‐Myc (Figure S4C,D, Supporting Information). IHC staining revealed that xenograft tumor tissues from the RNF112‐overexpressing group presented lower levels of c‐Myc protein than did those from the control group (Figure S4E, Supporting Information). Moreover, the paired BLCA and paracarcinoma tissues collected by our group further confirmed that the RNF112 protein was negatively correlated with the c‐Myc protein in both BLCA and paracarcinoma tissues (Figure S4F, Supporting Information).

**Figure 4:**
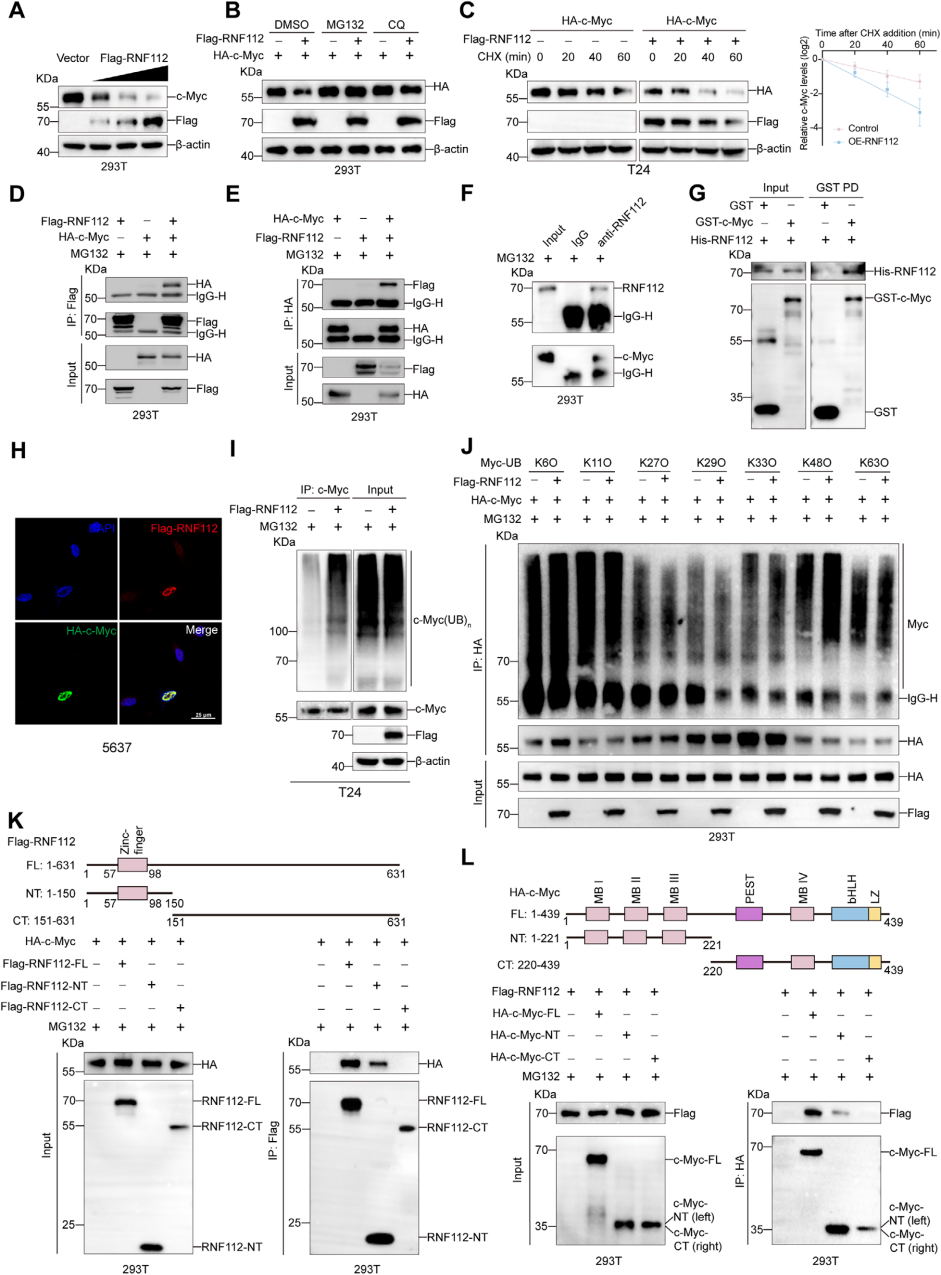
RNF112 interacts with c‐Myc and promotes its degradation by increasing its ubiquitination. A) 293T cells were transfected with graded concentrations of the RNF112 plasmid (0, 0.5, 1, or 2 µg) for 48 h for Western blot analyses. B) 293T cells were transfected with the respective plasmids and treated with DMSO, MG132 (10 µm), or CQ (25 µm) for 8 h for Western blot. C) Western blot analyses were conducted by transfecting the respective plasmids into T24 cells for 48 h and incubating them with CHX (50 µg mL^−1^) for the indicated times (*n* = 3). Representative images of the Western blot analyses (left) and the corresponding protein densitometry analysis (right) are shown. D,E) 293T cells were transfected with the respective plasmids for 48 h, and MG132 (10 µm) was added for 8 h. IP experiments were performed using anti‐Flag (D) or anti‐HA (E) antibodies. F) IP assay with RNF112 antibody in 293T cell lysates to verify the interaction between RNF112 and c‐Myc. G) A GST pull‐down assay was conducted to verify the direct interaction between RNF112 and the c‐Myc protein in vitro. H) IF assays were used to detect the expression and localization of Flag‐RNF112 (red) and HA‐c‐Myc (green) in 5637 cells, and the nuclei were stained with DAPI (blue). Scale bar = 25 µm. I) The respective plasmids were transfected into T24 cells for 48 h, and MG132 (10 µm) was added for 8 h. An anti‐c‐Myc antibody was added to perform the ubiquitination assays. J) The respective plasmids were transfected into 293T cells for 48 h, and MG132 (10 µm) was added for 8 h. Ubiquitination assays were performed to detect the specific type of ubiquitin chain linked to c‐Myc affected by RNF112. K,L) The respective plasmids were transfected into 293T cells for 48 h, and MG132 (10 µm) was added for 8 h. An anti‐Flag antibody (K) or anti‐HA antibody (L) was added for subsequent IP experiments.

In 293T cells, the protein level of c‐Myc decreased with increasing RNF112 concentration (**Figure** **4**A). Immunofluorescence staining revealed that RNF112 significantly reduced endogenous c‐Myc protein levels in BLCA cells (Figure S5A, Supporting Information). Treatment with MG132 reversed the decrease in c‐Myc protein levels caused by RNF112, suggesting that RNF112 may affect c‐Myc protein stability through the proteasome pathway (Figure 4B). CHX experiments demonstrated that overexpression of RNF112 promoted the degradation of the c‐Myc protein (Figure 4C). To further investigate the mechanism by which RNF112 regulates c‐Myc, we performed immunoprecipitation‐mass spectrometry (IP‐MS) analysis after overexpressing Flag‐RNF112 in bladder cancer T24 cells. The results revealed that c‐Myc was one of the interacting proteins of RNF112 (Figure S5B,C and Dataset S3, Supporting Information). Exogenous immunoprecipitation (IP) confirmed the interaction between RNF112 and c‐Myc (Figure 4D,E), which was also verified by endogenous IP (Figure 4F). Additionally, GST pulldown experiments demonstrated a direct interaction between RNF112 and c‐Myc (Figure 4G). Immunofluorescence staining revealed that RNF112 colocalized with c‐Myc in the nuclei of the 5637 cells (Figure 4H).

Given that RNF112 is an E3 ligase and that RNF112 may degrade c‐Myc via the proteasome pathway, we used ubiquitination experiments to examine the effect of RNF112 on the ubiquitination of the c‐Myc protein and revealed that RNF112 increased the ubiquitination level of the c‐Myc protein (Figure 4I; Figure S5D, Supporting Information).

The ubiquitin molecule can form a polyubiquitin chain through linkage at one of its seven lysine residues (Lys6, Lys11, Lys27, Lys29, Lys33, Lys48, and Lys63). The E3 ubiquitin ligase catalyzes the reaction, enabling the substrate to attach to the polyubiquitin chain, forming a polyubiquitinated substrate.^[^ ^16^ ^]^ To determine the specific type of ubiquitin chain involved, we constructed seven ubiquitin site mutant plasmids, including K48O (K48O ubiquitination refers to a ubiquitin construct in which all lysine residues, except for lysine 48, are mutated to arginine, allowing only the formation of K48‐linked ubiquitin chains). The results demonstrated that RNF112 promoted c‐Myc ubiquitination via K48‐linked ubiquitin chains (Figure 4J). After we transfected a ubiquitin plasmid with a K48R mutation (K48R involves the substitution of lysine 48 with arginine, preventing the formation of K48‐linked ubiquitin chains), RNF112 could not facilitate c‐Myc ubiquitination (Figure S5E,F, Supporting Information). This indicates that when K48‐linked ubiquitin chains cannot form, RNF112 is unable to promote the ubiquitination of c‐Myc. These results suggest that RNF112 promotes c‐Myc ubiquitination through K48‐linked ubiquitin chains.

**Figure 5:**
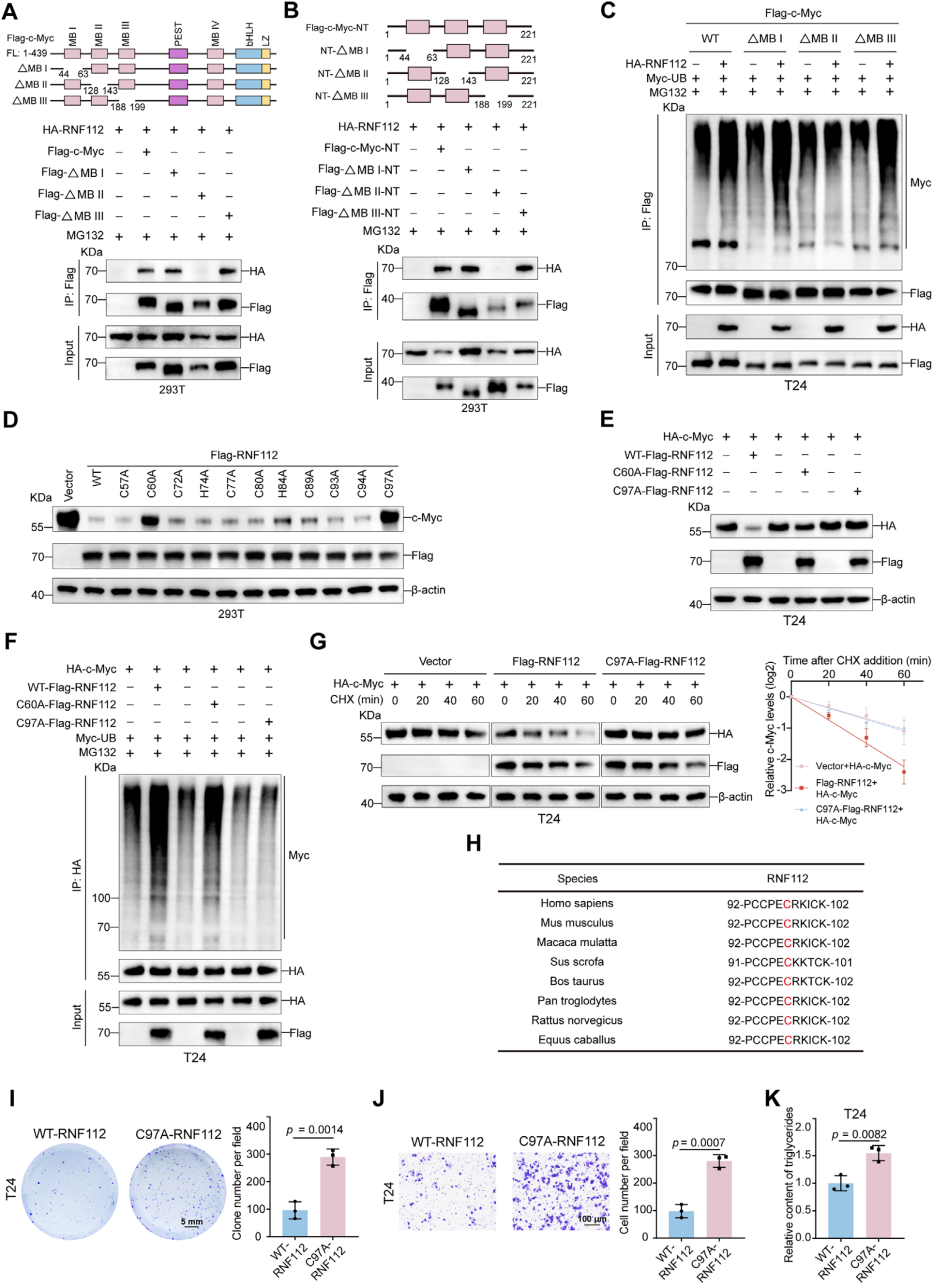
RNF112 binds to the MB II domain of c‐Myc and performs its E3 ligase function through the C97 site. A) The respective plasmids were transfected into 293T cells for 48 h, and MG132 (10 µm) was added for 8 h. An anti‐Flag antibody was added for IP experiments. B) The indicated plasmids were transfected into 293T cells for 48 h and MG132 (10 µm) was added for 8 h. Detection of key functional domains contained in the N segment of c‐Myc that interact with RNF112 via Co‐IP. C) The respective plasmids were transfected into T24 cells for 48 h, and MG132 (10 µm) was added for 8 h. Anti‐Flag antibody was added to perform the ubiquitination assays. D) 293T cells were transfected with the respective RNF112 mutant plasmids for 48 h, after which Western blot analysis was conducted to detect the expression of endogenous c‐Myc. E) The respective plasmids were transfected into T24 cells for 48 h, and a Western blot analysis was performed to detect the expression of exogenous HA‐c‐Myc. F) T24 cells were transfected with the respective plasmids for 48 h, and MG132 (10 µm) was added for 8 h. An anti‐HA antibody was added to perform the ubiquitination assays. G) Western blot analysis was conducted by transfecting the respective plasmids into T24 cells for 48 h and incubating them with CHX (50 µg mL^−1^) for the indicated times (*n* = 3). Representative images of the Western blot analyses (left) and the corresponding protein densitometry analysis (right). H) The amino acid sequence of RNF112 at the C97 site is highly conserved across multiple species. I) Representative images of plate colony formation assays after the overexpression of WT‐RNF112 or C97A‐RNF112 and the corresponding statistical graphs (*n* = 3) of T24 cells. Scale bar = 5 mm. J) Representative images of Transwell migration assays after the overexpression of WT‐RNF112 or C97A‐RNF112 and the corresponding statistical graphs (*n* = 3) of T24 cells. Scale bar = 100 µm. K) Statistical graphs showing the relative triglyceride content after the overexpression of WT‐RNF112 or C97A‐RNF112 in T24 cells. *p*‐values were determined by two‐tailed unpaired Student's *t*‐test (I‐K).

To identify the binding region of RNF112 on c‐Myc, we truncated the c‐Myc and RNF112 proteins into their N and C segments, respectively, on the basis of their structural domains. Coimmunoprecipitation (Co‐IP) experiments in 293T cells revealed that the N segment of RNF112 interacted with the N segment of c‐Myc (Figure 4K,L). The N segment of c‐Myc contains three functional domains: MB I, MB II, and MB III. We constructed plasmids with deletions of these domains in both the wild‐type c‐Myc protein and the N‐truncated protein and then performed Co‐IP experiments. Deletion of the MB II domain in both the wild‐type and N‐truncated c‐Myc proteins prevented RNF112 from interacting with c‐Myc (**Figure** **5**A,B). We subsequently cotransfected full‐length c‐Myc plasmids with deletions in the MB I (ΔMB I), MB II (ΔMB II), and MB III (ΔMB III) domains with the RNF112 plasmid and performed ubiquitination and Western blot analyses. RNF112 did not increase the ubiquitination level of c‐Myc or decrease its total protein level in the ΔMB II group (Figure 5C; Figure S5G,H, Supporting Information). These findings indicate that RNF112 binds to the MB II domain of the c‐Myc protein and affects its ubiquitinated degradation.

### 2.5 C97 Provides the E3 Ubiquitinase‐Activating Function of RNF112 and Promotes the Ubiquitination of c‐Myc at K389

To determine the functional site of the enzymatic activity of RNF112, we mutated all cysteines and histidines in its RING‐finger domain to alanines, constructing a total of 11 RNF112 mutant plasmids (C57A, C60A, C72A, H74A, C77A, C80A, H84A, C89A, C93A, C94A, and C97A). After each mutant RNF112 expression plasmid was overexpressed in 293T cells, we detected c‐Myc via Western blot. Only the C60A and C97A mutants failed to reduce the protein level of c‐Myc (Figure 5D). This result was verified in T24 cells, where the C60A mutation led to partial recovery of c‐Myc levels, but the C97A mutation resulted in complete recovery of c‐Myc protein expression (Figure 5E). Subsequent ubiquitination experiments revealed that the C97A mutation in RNF112 did not increase the level of c‐Myc ubiquitination, whereas the C60A mutation still increased c‐Myc ubiquitination to some extent (Figure 5F; Figure S6A, Supporting Information). After T24 cells were transfected with the wild‐type RNF112 plasmid or the C97A‐RNF112 plasmid, c‐Myc stability was restored in the C97A‐RNF112 group compared with that in the wild‐type RNF112 group (Figure 5G). The importance of this locus was further confirmed by gene sequence comparison, which revealed that C97 in RNF112 was conserved across multiple species (Figure 5H). To determine whether the enzyme‐activated locus plays a role in the phenotype of BLCA cells, we overexpressed both wild‐type RNF112 (WT‐RNF112) and the inactivation mutant RNF112 (C97A‐RNF112) in BLCA cells (T24 and 5637). The results revealed a significant increase in the proliferation and migration ability of BLCA cells in the C97A‐RNF112 group compared with those in the WT‐RNF112 group (Figure 5I,J; Figure S6B,C, Supporting Information). Furthermore, the triglyceride content was greater in the C97A‐RNF112 group than in the WT‐RNF112 group (Figure 5K; Figure S6D, Supporting Information). The above findings demonstrate that C97 is a key site of RNF112 enzymatic activity.

To determine the exact ubiquitination site at which RNF112 targets c‐Myc, we mutated 25 lysine residues in the coding sequence region of c‐Myc to arginine. The RNF112 plasmid was cotransfected with these 25 c‐Myc mutant plasmids in 293T cells, and the gray values of the bands of the different c‐Myc mutant groups were detected and ranked by Western blot analysis (Figure S7A,B, Supporting Information). The top seven groups (K52R, K275R, K341R, K389R, K392R, K397R, and K430R) were selected for a second round of validation in T24 cells according to the relative expression levels of c‐Myc proteins. Western blot analysis revealed that c‐Myc protein levels remained unchanged in the K341R, K389R, and K430R groups compared with those in the other groups (Figure S7C,D, Supporting Information). We subsequently cotransfected the K341R, K389R, and K430R c‐Myc plasmids with the RNF112 plasmid and performed ubiquitination experiments. RNF112 did not increase the level of ubiquitinated c‐Myc protein in the K389R group (Figure S7E, Supporting Information). Finally, the CHX assay demonstrated that the half‐life of c‐Myc in the K389R group was significantly longer than that in the wild‐type c‐Myc group (Figure S7F,G, Supporting Information). The above experimental results indicate that RNF112 targets and promotes ubiquitination at the K389 locus of c‐Myc.

### 2.6 RNF112 Inhibits BLCA Progression via c‐Myc Both In Vitro and In Vivo

**Figure 6:**
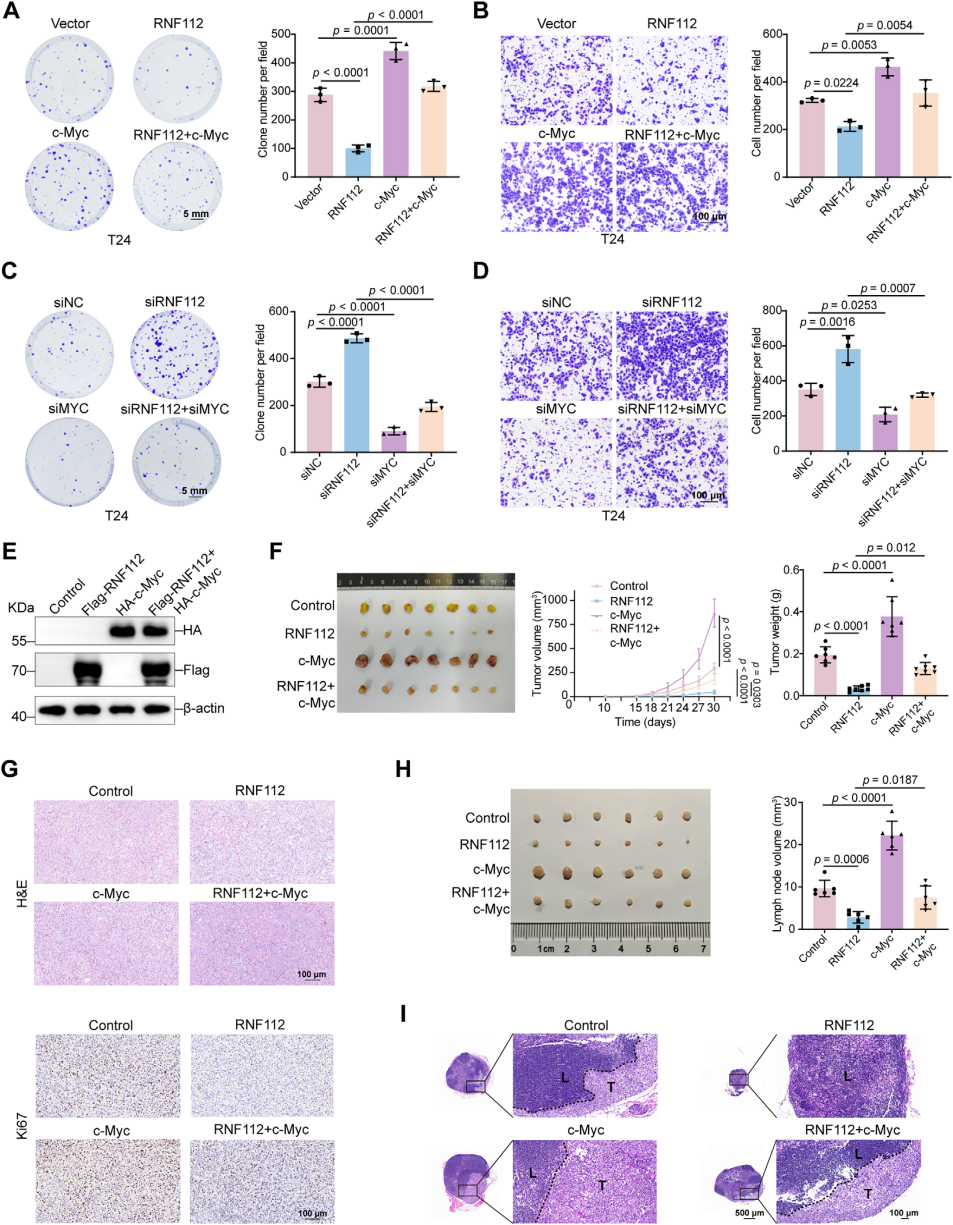
RNF112 inhibits BLCA progression via c‐Myc both in vitro and in vivo. A) Representative images and statistical analysis of colony formation assays in T24 cells overexpressing RNF112 and c‐Myc (*n* = 3). Scale bar = 5 mm. B) Representative images and statistical analysis of transwell migration assays in T24 cells overexpressing RNF112 and c‐Myc (*n* = 3). Scale bar = 100 µm. C) Representative images and statistical analysis of colony formation assays in T24 cells with RNF112 and c‐Myc knockdown (*n* = 3). Scale bar = 5 mm. D) Representative images and statistical analysis of Transwell assays in T24 cells with RNF112 and c‐Myc knockdown (*n* = 3). Scale bar = 100 µm. E) Western blot analysis to verify the successful establishment of four stably overexpressing T24 cell lines (Control, Flag‐RNF112, HA‐c‐Myc, and Flag‐RNF112+HA‐c‐Myc). F) Gross images of subcutaneous tumor tissues (left) and corresponding volume (center) and weight statistics (right) from xenograft models constructed from four groups (Control, RNF112, c‐Myc, and RNF112+ c‐Myc) of T24 cells (*n* = 7). G) Representative images of H&E staining (top) and Ki67 staining (bottom) of four sets of subcutaneous tumor tissue. Scale bar = 100 µm. H) Gross image of popliteal lymph nodes (left) and corresponding volume statistical graph (right) in four stable overexpression groups (Control, RNF112, c‐Myc, and RNF112+ c‐Myc) constructed from T24 cells (*n* = 6). I) Representative images of H&E‐stained popliteal lymph nodes from the four groups (Control, RNF112, c‐Myc, and RNF112+ c‐Myc). Scale bar = 500 µm (left). L = lymphoid tissue; T = metastatic tumor. Scale bar of the enlarged images = 100 µm (right). *p*‐values were determined by one‐way ANOVA followed by Dunnett's multiple comparisons test (A, B, C, D, F, and H).

To confirm whether RNF112 inhibits the proliferation and metastasis of BLCA through c‐Myc, we performed plate colony formation assays and transwell migration assays after knockdown or overexpression of RNF112 and c‐Myc, respectively, or simultaneously, in BLCA cells (T24 and 5637). The results confirmed that simultaneous overexpression of c‐Myc and RNF112 rescued the inhibitory effect of RNF112 on the proliferation and migration of T24 and 5637 cells (**Figure** **6**A,B; Figure S8A,B, Supporting Information), whereas simultaneous knockdown of RNF112 and c‐Myc attenuated the promoting effect of RNF112 knockdown on the proliferation and migration of BLCA cells (Figure 6C,D; Figure S8C,D, Supporting Information). In addition, overexpression of RNF112 resulted in a significant decrease in the expression of proliferation‐associated proteins (Cyclin E1, Cyclin D1, and PCNA) and EMT‐associated proteins (Vimentin, Snail, and N‐cadherin) and an increase in the expression of E‐cadherin, which was consistent with previous results, and simultaneous overexpression of c‐Myc and RNF112 reversed these changes (Figure S8E, Supporting Information). Conversely, RNF112 knockdown led to significant upregulation of proliferation‐associated proteins and EMT‐associated proteins (Vimentin, Snail, and N‐cadherin), which was largely reversed by concurrent knockdown of c‐Myc (Figure S8F, Supporting Information). Taken together, these results indicate that RNF112 inhibits BLCA proliferation and migration in vitro by downregulating c‐Myc.

To further validate these findings in vivo, we established four stably transduced cell lines with T24 cells: LV‐Control (Control), LV‐RNF112 (RNF112), LV‐c‐Myc (c‐Myc), and LV‐RNF112+c‐Myc (RNF112+c‐Myc) (Figure 6E). The subcutaneous xenograft model confirmed a significant reduction in tumor volume and weight in the RNF112 group compared with those in the control group. Compared with those in the RNF112 group, the tumor volume and weight in the RNF112+c‐Myc group were significantly greater (Figure 6F). H&E staining and Ki67 staining of subcutaneous tumor tissues revealed a significant increase in heterogeneity and proliferative capacity in the RNF112+c‐Myc group compared with the RNF112 group (Figure 6G). Additionally, the popliteal lymphatic metastasis model revealed that the volume of popliteal lymph nodes was significantly lower in the RNF112 group than in the control group but was significantly greater in the RNF112+c‐Myc group than in the RNF112 group (Figure 6H). H&E staining of the lymph nodes confirmed that more extensive tumor metastasis occurred in the lymph nodes of the RNF112+c‐Myc group than in those of the RNF112 group (Figure 6I). The above in vivo results confirmed that RNF112 affects BLCA growth and metastasis through c‐Myc, which is consistent with the in vitro results.

### 2.7 RNF112 Inhibits Lipid Synthesis in BLCA Cells by Regulating ACLY Transcription via c‐Myc

**Figure 7:**
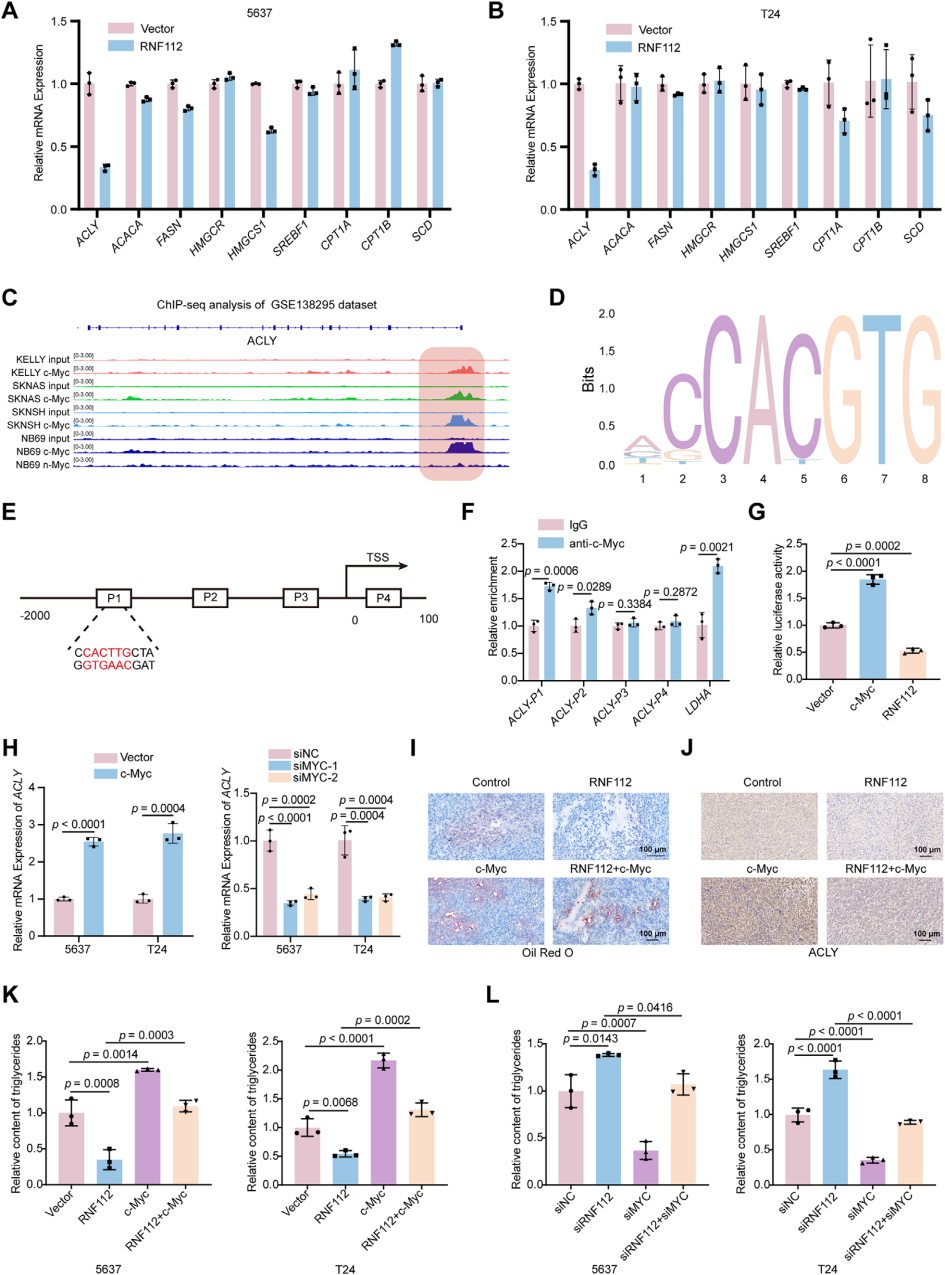
RNF112 inhibits lipid synthesis in BLCA cells by attenuating the transcriptional level of *ACLY* via the transcription factor c‐Myc. A,B) The mRNA levels of key lipid synthases and related transcription factors in 5637 (A) and T24 (B) cells after RNF112 overexpression were analyzed via qRT‐PCR (*n* = 3). C) The GSE138295 dataset was used to visualize c‐Myc or n‐Myc occupancy at *ACLY* loci via IGV software. D) Prediction of the motif sequence of *MYC* via the JASPAR database. E) A schematic diagram illustrating the three binding sites on the *ACLY* promoter was created on the basis of information from the JASPAR website. The P4 group served as the negative control. F) ChIP‐qPCR was performed to analyze the enrichment of c‐Myc at the *ACLY* promoter sequence in 5637 cells (*n* = 3). G) In 293T cells, c‐Myc increased the promoter luciferase activity of *ACLY*, whereas RNF112 suppressed the promoter luciferase activity of *ACLY*. The luciferase activity of the reporter gene was normalized to that of Renilla luciferase (*n* = 3). H) qRT‐PCR was used to assess the effects of *MYC* knockdown or overexpression on *ACLY* mRNA levels in the 5637 and T24 cells (*n* = 3). I,J) Representative images of Oil Red O staining (I) and IHC staining (J) of tumor tissue from four groups of mice (Control, RNF112, c‐Myc, and RNF112+ c‐Myc). Scale bar = 100 µm. K) Statistical graphs showing the relative triglyceride levels in the 5637 and T24 cells overexpressing RNF112 and c‐Myc. L) Statistical graphs showing the relative triglyceride levels in the 5637 and T24 cells in which RNF112 and c‐Myc were knocked down. *p*‐values were determined by two‐tailed unpaired Student's *t*‐test (F and H‐left) and one‐way ANOVA with Dunnett's multiple comparisons (G, H‐right, K, and L).

To investigate the specific mechanism by which RNF112 inhibits lipid synthesis, we examined the changes in the mRNA expression of several lipid metabolism‐related enzymes and transcription factors, including SREBF1 (a key transcription factor that regulates lipid metabolism), ACLY (a key enzyme that regulates the synthesis of acetyl‐CoA), key cholesterol synthesis enzymes (HMGCR and HMGCS1), key fatty acid synthesis enzymes (ACACA, FASN, and SCD1), and key fatty acid oxidation enzymes (CPT1A and CPT1B). The results revealed that the mRNA level of *ACLY* was significantly downregulated in both BLCA cell lines (5637 and T24) overexpressing RNF112 (**Figure** **7**A,B). Further Western blot analyses demonstrated that the overexpression of RNF112 decreased the ACLY protein level, whereas the knockdown of RNF112 increased the ACLY protein level (Figure S9A,B, Supporting Information). Previous studies have reported that c‐Myc can directly regulate the transcription of key enzymes involved in lipid synthesis. Analysis of the GSE138295 dataset^[^ ^17^ ^]^ revealed that c‐Myc, but not n‐Myc, has a significant signal in the promoter region of *ACLY* (Figure 7C). Additionally, the GEPIA dataset revealed a positive correlation between *MYC* and *ACLY* (Figure S9C, Supporting Information). On the basis of these findings, we hypothesized that c‐Myc may bind to the promoter region of *ACLY*, thereby promoting its transcription. We queried possible c‐Myc binding sequences in the *ACLY* promoter region via the JASPAR database (Figure 7D). On the basis of the presence of the CANNTG sequence,^[^ ^18^ ^]^ we designed three predicted binding regions and one negative control region (Figure 7E). The results of the ChIP‐qPCR assay confirmed that c‐Myc could directly bind to the P1 segment of the *ACLY* promoter region in 5637 cells (Figure 7F). We then constructed a plasmid containing the firefly luciferase reporter gene driven by the *ACLY* promoter. The dual‐luciferase reporter assay revealed that c‐Myc promoted the transcriptional activity of *ACLY*, whereas RNF112 inhibited the transcriptional activity of *ACLY* (Figure 7G). Consistently, knockdown or overexpression of c‐Myc in 5637 and T24 cells resulted in the corresponding downregulation or upregulation of *ACLY* mRNA, respectively (Figure 7H; Figure S9D,E, Supporting Information). In addition, we performed Oil Red O staining and IHC staining for ACLY in tumor tissue from the subcutaneous xenograft model and revealed significantly reduced lipid droplet content and ACLY expression in the xenograft tissues of the RNF112 group. Moreover, compared with those in the RNF112 group, the lipid droplet content and ACLY expression were greater in the RNF112+c‐Myc group (Figure 7I,J). After the intracellular triglyceride content in each group was detected, we found that RNF112 significantly decreased the intracellular triglyceride content, whereas c‐Myc overexpression completely reversed the effect of RNF112 on the triglyceride content in BLCA cells (Figure 7K,L). These findings indicate that RNF112 inhibits lipid synthesis in BLCA cells by attenuating the transcriptional regulation of *ACLY* via the transcription factor c‐Myc.

To determine whether RNF112 regulates the proliferation, metastasis, and lipid synthesis of BLCA cells via ACLY, we performed plate colony formation assays, transwell migration assays, and triglyceride content measurements after RNF112 and ACLY were individually or simultaneously overexpressed in BLCA cells (T24 and 5637). The results revealed that simultaneous overexpression of ACLY and RNF112 successfully reversed the inhibitory effects of RNF112 overexpression alone on cell proliferation, migration, and lipid synthesis (Figure S10A–F, Supporting Information). Conversely, knockdown of RNF112 combined with treatment with the ACLY inhibitor SB 204990 significantly diminished the promoting effects of RNF112 knockdown on these processes (Figure S10G–L, Supporting Information). These findings emphasize the pivotal role of ACLY in RNF112‐mediated BLCA progression. To further validate the relationship between ACLY and c‐Myc, we conducted rescue experiments in BLCA cell lines. The results showed that the overexpression of c‐Myc in conjunction with ACLY inhibition successfully reversed the promoting effects of c‐Myc on proliferation, migration, and lipid synthesis (Figure S11A–F, Supporting Information). Conversely, knocking down c‐Myc while overexpressing ACLY significantly reversed the inhibitory effects of c‐Myc knockdown on these phenotypes (Figure S11G–L, Supporting Information).

## 3 Discussion

**Figure 8:**
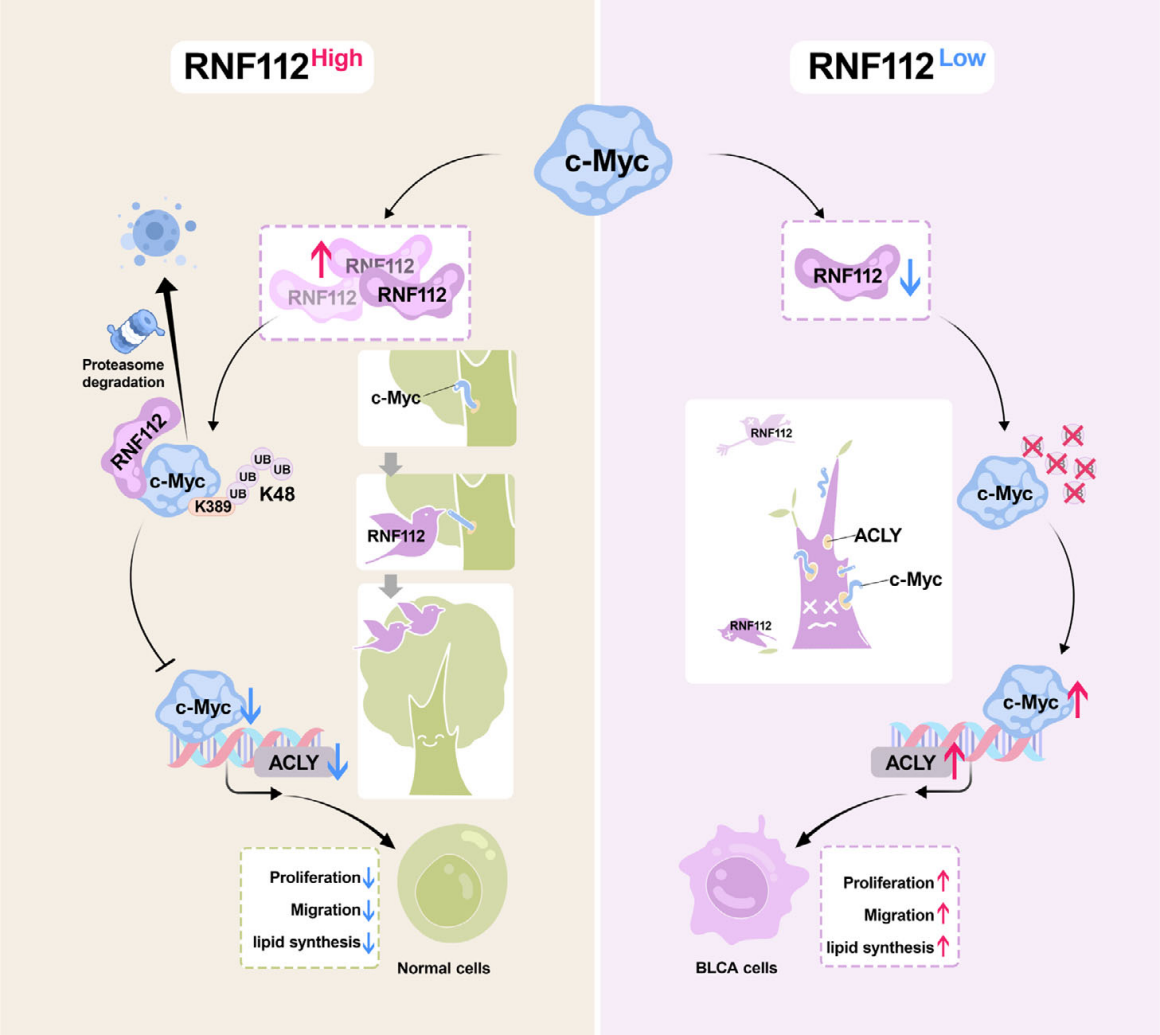
Mechanistic diagram of this study. The E3 ligase RNF112 directly interacts with c‐Myc through its N‐terminal zinc finger motif and catalyzes K48‐linked polyubiquitination at K389 on c‐Myc, facilitating its degradation. Furthermore, c‐Myc can increase the transcriptional activity of *ACLY* by binding to its promoter region. Ultimately, RNF112 affects lipid synthesis, proliferation, and metastasis via the c‐Myc/ACLY axis in BLCA cells.

Few previous studies have explored the role and function of RNF112 in tumors. In our study, we found that RNF112 is downregulated in BLCA tissues and cells, which may be associated with a high frequency of *RNF112* mutations or deep deletions in BLCA. A series of in vitro and in vivo experiments revealed that the overexpression of RNF112 suppresses the growth and migration of BLCA cells. Through RNA‐seq analysis and mechanistic studies, we subsequently revealed that RNF112 promotes the ubiquitination and degradation of c‐Myc proteins, whereas c‐Myc regulates downstream *ACLY* transcription, which ultimately affects lipid metabolism and the malignant phenotype of BLCA cells (**Figure** **8**).

Notably, the *RNF112* mRNA level did not decrease markedly with BLCA progression but rather remained consistently low in BLCA. As RNF112 is an oncogene in BLCA, the low level of *RNF112* mRNA expression may be the reason for its difficulty in detecting statistically significant differences in different stages of BLCA. In contrast, the protein level of RNF112 was significantly lower in late‐stage tissues or MIBC tissues than in early‐stage tissues or NMIBC tissues. The discrepancies between the *RNF112* mRNA and protein levels warrant further investigation.

Studies have reported that nearly all patients with locally advanced BLCA exhibit *MYC* amplification,^[^ ^19^ ^]^ and early‐stage BLCA with abnormally high *MYC* expression is associated with a higher recurrence rate.^[^ ^20^ ^]^ Owing to the lack of an enzymatic pocket, c‐Myc is difficult to target with small‐molecule drugs.^[^ ^21^ ^]^ Given the importance of c‐Myc in BLCA and its degradation via the ubiquitination pathway, exploration of E3 ligases upstream of c‐Myc has been ongoing. Many E3 ligases, such as FBXW7, Skp2, HectH9, Fbx29, and TRAIP, have been reported to interact with c‐Myc.^[^ ^5^, ^22^ ^]^ Our study revealed that the N‐terminal zinc finger domain of RNF112 interacts with the MB II domain of the c‐Myc protein. Previous reports indicate that Skp2 and TRAIP also bind to the MB II domain of the c‐Myc protein and promote its ubiquitination.^[^ ^5^, ^22^ ^]^ We hypothesize that interactions between different E3 ligases may form complexes, which warrants further investigation. By mutating the lysine residues of c‐Myc, we discovered that RNF112 primarily catalyzes the ubiquitination of the K389 residue on c‐Myc, a site that has also been reported to be sumoylated via mass spectrometry.^[^ ^23^ ^]^ However, not all E3 ligases ubiquitinate c‐Myc at the same lysine residue; for instance, TRAIP primarily ubiquitinates c‐Myc at K428/430. For the first time, we showed that RNF112 exerts its E3 ligase function through its C97 residue. Additionally, mutation of the C60 residue of RNF112 partially impairs its E3 ligase activity, suggesting that RNF112 may have multiple active sites, with the C97 residue being the most critical.

c‐Myc is involved in reprogramming lipid metabolism in various tumors.^[^ ^24^ ^]^ However, its role in lipid metabolism in BLCA remains unclear. Previous studies have shown that c‐Myc can interact with the promoter of SREBP1, resulting in the reprogramming of fatty acid metabolism in liver cancer cells.^[^ ^14^ ^]^ Moreover, c‐Myc has been demonstrated to increase the synthesis of particular eicosanoids in lung cancer cells, promoting the progression of lung cancer.^[^ ^24b^ ^]^ In B‐cell lymphoma cell lines, c‐Myc stimulates increases in the mRNA and protein levels of crucial lipid synthesis enzymes, including ACACA, FASN, and SCD1. Additionally, c‐Myc has been shown to bind to the promoters of the majority of fatty acid synthesis genes to promote lipid synthesis.^[^ ^14^ ^]^ A decrease in the number of intracellular lipid droplets and triglyceride content resulting from RNF112 overexpression was noted. On the basis of our previous findings, which established a relationship between RNF112 and c‐Myc and demonstrated that RNF112 significantly reduces the intracellular lipid content, we hypothesized that RNF112 affects the lipid metabolism of BLCA by modulating c‐Myc activity. Previous studies have reported that the transcription factor c‐Myc plays a critical role in regulating lipid metabolism.^[^ ^14^ ^]^ On the basis of our findings and previous studies,^[^ ^25^ ^]^ we selected several representative key enzymes and transcription factors (SREBF1, ACLY, HMGCR, HMGCS1, ACACA, FASN, SCD1, CPT1A, and CPT1B) associated with lipid metabolism for subsequent studies and found that only the mRNA expression of *ACLY* significantly decreased after the overexpression of RNF112 in BLCA cells. ChIP and dual‐luciferase reporter assays confirmed that in BLCA cells, c‐Myc directly binds to the promoter of *ACLY*, increasing its transcriptional activity, whereas RNF112 suppresses the transcriptional activity of *ACLY*. Although RNF112 affects c‐Myc protein stability, it remains to be determined whether it affects *ACLY* transcriptional activity through other mechanisms. Additionally, whether RNF112 affects lipid synthesis in BLCA through lipid metabolism molecules other than ACLY warrants further investigation.

Lipid metabolism reprogramming is a common occurrence in aggressive tumors, such as BLCA, where essential lipid synthesis enzymes such as ACLY and FASN are upregulated.^[^ ^26^ ^]^ Lipid droplets are unique intracellular organelles that not only store energy but also relieve endoplasmic reticulum stress and lipotoxic stress.^[^ ^27^ ^]^ Therefore, targeting lipid synthesis in tumors is considered a promising cancer therapeutic strategy. ACLY, a pivotal enzyme involved in de novo lipid synthesis, catalyzes the conversion of citrate to acetyl‐CoA, thereby supplying the necessary substrate for the biosynthesis of fatty acids and cholesterol.^[^ ^26^, ^28^ ^]^ The overexpression of RNF112 in BLCA cells resulted in a decrease in the intracellular lipid droplet and triglyceride content, which may be related to the downregulation of ACLY. Nevertheless, additional research is necessary to ascertain whether RNF112 suppresses lipid biosynthesis in BLCA via ACLY and to assess the viability of ACLY as a potential therapeutic target for BLCA.

In conclusion, our study revealed that RNF112 inhibits the growth and metastasis of BLCA through its E3 ligase activity at its N‐terminal zinc finger structural domain C97 site. Specifically, RNF112 promotes K48‐linked polyubiquitination at the K389 lysine residue of the c‐Myc protein, leading to degradation of the c‐Myc protein. Furthermore, c‐Myc can increase the transcriptional activity of *ACLY* by binding to its promoter region. RNF112 leads to a decrease in intracellular lipid droplets and triglycerides and inhibits the proliferation and metastasis of BLCA cells by suppressing the expression of c‐Myc (Figure 8).

## 4 Experimental Section

### Tissue Samples

The research was carried out with the endorsement of the Medical Ethics Committee of Zhongnan Hospital of Wuhan University (approval number: 2022177K). Human BLCA tissues and paired adjacent tissues were collected from Zhongnan Hospital of Wuhan University with informed consent. The clinicopathological features of the BLCA patients are detailed in Table S2 (Supporting Information). BLCA tissue microarrays (HBlaU108Su01) were obtained from Outdo Biotech (Suzhou, China).

### Cell Line Culture

The cell lines were obtained from the cell bank of the Chinese Academy of Science and verified through short tandem repeat (STR) profiling. SV‐HUC‐1, 5637, SCaBER, and T24 cells were cultured in RPMI 1640 medium. MEM was used to culture UM‐UC‐3 and J82 cells. The RT4 cells were cultured in McCoy's 5A. DMEM was used to culture the 293T cells. To each medium, 10% fetal bovine serum and 1% penicillin/streptomycin were added. The cells were incubated at 37 °C with 5% CO_2_ in culture.

### siRNAs and Plasmids

The siRNAs used for this study were purchased from GenePharma (Suzhou, China). The siRNA sequences are listed in Table S3 (Supporting Information).

Professor Guoliang Qing generously provided the HA‐c‐Myc and Flag‐c‐Myc plasmids as gifts. The plasmid Flag‐RNF112 was purchased from Miaoling Biology (Wuhan, China). All remaining plasmids were constructed via molecular cloning, and DNA sequencing was performed to confirm the correctness of the plasmid sequences. The transfection procedures for both the siRNAs and the plasmids were carried out as per the guidelines provided for the Lipofectamine TM 3000 Reagent (Cat. #L3000075; Invitrogen).

### RNA Extraction and Quantitative Reverse Transcription PCR (qRT‐PCR)

RNA extraction, reverse transcription, and qRT‐PCR were performed as described previously.^[^ ^29^ ^]^ The primers used are listed in Table S4 (Supporting Information).

### RNA Sequencing (RNA‐seq)

RNA extraction was performed with TRIzol reagent following the guidelines provided by the manufacturer. Shanghai Ouyi Biotechnology Co., Ltd. conducted transcriptome sequencing and analysis. The RNA‐seq data from this study had been deposited in the Gene Expression Omnibus (GEO) database with the accession number GSE270143.

### Colony Formation Assay

After counting, the treated cells were placed in 6‐well plates with 1000 cells in each well for culture. The medium was changed after 3 days of cell adherence. After 1–2 weeks, the cells were treated with 4% paraformaldehyde for 2 h, followed by staining with 800 µL of 0.1% crystal violet solution per well for an additional 2 h. The plates were subsequently washed in distilled water, dried, photographed and counted via Image‐Pro Plus.

### CCK8 Cell Proliferation Assay

After treatment, the cells were placed in 96‐well plates with 2000 cells in each well. Once the cells had adhered to the plate, the medium was replaced with fresh medium supplemented with 10% CCK‐8 solution from days 1 to 5. After the CCK‐8 solution was added, the cells were incubated for 2 h. A microplate reader was subsequently used to measure the optical density (OD) at 450 nm.

### Wound Healing Assay

The treated cells were inoculated in 6‐well plates and allowed to grow to 90% confluence. Straight scratches were made via 200 µL micropipette tips. The cells were then incubated in serum‐free medium for 24 h. Images were taken at the same sites both immediately after scratching and after the 24 h incubation period to assess cell migration. Migration rate (%) = (1 – 24 h scratch distance/0 h scratch distance) × 100%.

### Transwell Migration Assay

Six hundred microliters of complete medium was added to the lower chamber. A total of 3 × 10^4^ T24 cells or 7 × 10^4^ 5637 cells were resuspended in 100 µL of serum‐free medium and added to the top transwell chambers. After 24 h of incubation, the chambers were fixed with 4% paraformaldehyde for 2 h and stained with 0.1% crystal violet solution for 1 h. The chambers were washed with distilled water to remove the crystal violet dye and then dried and photographed.

### IP‐MS Analysis

T24 cells were transfected with Flag‐RNF112 overexpression plasmid for two days, after which the cells were collected for IP assays and subsequent protein separation by SDS‐PAGE. The gel was silver stained according to the instructions of the silver staining kit (Cat. #P0017S, Beyotime). Mass spectrometry analysis and data reanalysis were performed by Shanghai Luming Biological Technology Co., Ltd. (Shanghai, China).

### Liquid Chromatography‐Mass Spectrometry (LC‐MS)‐Based Untargeted Metabolomic Analysis

T24 cells were transfected with empty plasmid or RNF112 overexpression plasmid for two days, after which the cells were collected for subsequent assays. LC‐MS‐based untargeted metabolomics analyses and data reanalysis were performed by Shanghai Luming Biological Technology Co., Ltd. (Shanghai, China). Metabolites were considered significantly different if they met the criteria of *p*‐value < 0.05 and a fold change (FC) ≥ 1.2 or ≤ 1/1.2.

### Triglyceride Quantification

The cells were collected and then suspended in 150 µL of PBS. The cells were then lysed by sonication on ice. The protein concentration and triglyceride content of the lysates were measured. The protein concentration was measured with a BCA protein assay kit (Cat. #P0012, Beyotime). Triglycerides in cells were quantified with a triglyceride measurement kit (Cat. #A110, Nanjing Jiancheng) according to the provided guidelines. The relative triglyceride content was calculated by normalizing the triglyceride content to the protein concentration.

### Oil Red O Staining

Treated cells were plated on cell plates, fixed in 4% paraformaldehyde and stained with Oil Red O following established protocols. Cryosections of subcutaneous tumor‐bearing tissue embedded in optimal cutting temperature (OCT) compound were stained with Oil Red O via a standard protocol.

### Western Blot Analyses

Briefly, the cells were lysed via RIPA buffer (containing PMSF and phosphatase inhibitors) on ice for 1 h. After lysis, 5 × loading buffer was added to the lysates, and the proteins were denatured by heating at 95 °C for 15 min. The proteins were separated and identified via methods outlined in a previous study. ^[^ ^12a^ ^]^ Protein quantification was performed via Image Lab software. The primary antibodies used in the study are listed in Table S5 (Supporting Information).

### Co‐IP Assays and Ubiquitination Assays

For Co‐IP assays, cells transfected with the corresponding plasmids were lysed with IP buffer for 1 h at 4 °C. The lysate was incubated with the appropriate primary antibodies overnight at 4 °C. The following day, clean magnetic beads were introduced to the samples, which were then incubated for another 2 h. The beads were cleansed following the guidelines provided by the Immunoprecipitation Kit (Cat. #22202‐100, Beaver). After washing, 25 µL of 1 × loading buffer was added to extract the proteins. The beads were subsequently heated to 100 °C for 10 min in preparation for Western blot analysis.

To conduct the ubiquitination test, cells that had been transfected with the appropriate plasmids were treated with 10 µm MG132 for 8 h prior to being collected. The cells were then lysed on ice and divided into two groups: input and IP. The corresponding labeled antibodies were added to each group. The ubiquitinated protein content was subsequently detected following the procedures described for the IP and Western blot assays.

### GST Pull‐Down Assay

Recombinant GST, GST‐c‐Myc, and His‐RNF112 proteins were purchased from CUSABIO Biotech Co., Ltd. For GST pull‐down, 2 µg of His‐RNF112 protein was incubated with 2 µg of either GST or GST‐c‐Myc protein in IP buffer for 4 h at 4 °C. Following this incubation, an additional 4 h of incubation were performed after the addition of 50 µL of glutathione‐Sepharose beads. The beads were washed three times with IP buffer, and 25 µL of 1 × loading buffer was added. The beads were subsequently heated to 100 °C for 10 min in preparation for Western blot.

### Chromatin Immunoprecipitation (ChIP) Assay

The ChIP assay was conducted with a ChIP assay kit (Cat. #56 383; Cell Signaling Technology) following the manufacturer's instructions. Briefly, 10 million cells were treated with 1% formaldehyde for 10 min, followed by the addition of glycine (0.125 m) for 5 min to stop the reaction. After being treated with ChIP lysis buffer on ice for 1 h, the cells were then sonicated to shear the DNA fragments. After the lysates were divided into IgG and IP groups by the addition of either IgG (Cat. #B900610, Proteintech) or anti‐c‐Myc antibody (Cat. #ab32072, Abcam), they were incubated overnight at 4 °C. The lysates were subsequently incubated with washed magnetic beads for 4 h. Afterward, the beads were rinsed three times with low‐salt and high‐salt solutions, and the DNA fragments were released via elution buffer. Afterward, the refined DNA fragments were subjected to quantitative PCR (qPCR). The ChIP primers used are listed in Table S6 (Supporting Information).

### Dual‐Luciferase Reporter Assays

The corresponding luciferase plasmids were transfected into 293T cells for 48 h. The Dual‐Luciferase Reporter Assay System (Cat. #E1910, Promega) was used to perform the assay, with Renilla luciferase used as a control to monitor transfection efficiency.

### Hematoxylin‐Eosin (H&E)

After formalin fixation, the tissue samples were dehydrated, paraffin‐embedded, and stained with HE according to standard procedures.

### Immunohistochemistry (IHC)

After formalin fixation, the tissue samples were dehydrated and then embedded in paraffin. The samples were then sectioned, dewaxed, subjected to antigen retrieval, and blocked with 5% BSA. After incubation with primary and secondary antibodies, a color reaction was performed using DAB. Images of the sections were ultimately acquired with a scanning microscope (Aperio VERSA 8, Leica). Table S5 (Supporting Information) contains a list of the primary antibodies that were utilized.

### Immunofluorescence (IF)

After treatment, the cells were placed on slides and allowed to attach to the surface before being treated with 4% paraformaldehyde. The cell membranes were permeabilized with 0.4% Triton X‐100, followed by blocking with 2% BSA. The cells were then incubated with primary and secondary antibodies in the dark. The nuclei were labeled with 0.5 µg mL^−1^ DAPI. Finally, images were acquired via a laser confocal microscope (C2^+^, Nikon).

### Animal Experiments

Flag‐RNF112 and HA‐c‐Myc lentiviruses were purchased from GenePharma (Suzhou, China). T24 cells were infected with these lentiviruses, and stably transfected cell lines were selected with 1 µg mL^−1^ puromycin 48 h postinfection. The establishment of RNF112‐ and c‐Myc‐stably overexpressing cell lines was confirmed by qRT‐PCR and Western blot analyses. The animal experiments in this study were approved by the Animal Welfare Ethics Committee of Zhongnan Hospital of Wuhan University (approval number: ZN2023232). Four‐week‐old male BALB/c nude mice were purchased from Beiente Biotechnology (Wuhan, China) and acclimatized to a barrier environment for one week.

To examine the impact of RNF112 on the proliferation and metastasis of BLCA in vivo, the mice were randomly allocated into two groups (the control group and the stable RNF112 overexpression group) in both a subcutaneous xenograft model (*n* = 8) and a footpad‐popliteal lymph node metastasis model (*n* = 6). To examine the potential dependence of RNF112‐induced inhibition of BLCA progression on c‐Myc, the mice were randomly allocated into four groups (the control group, the stable RNF112 overexpression group, the stable c‐Myc overexpression group, and the stable RNF112+c‐Myc overexpression group) for both subcutaneous xenograft reversion experiments (*n* = 7) and popliteal lymph node metastasis reversion experiments (*n* = 6).

For the subcutaneous xenograft model, after the subcutaneous injection of 1 × 10^7^ cells, the mice were observed and measured every three days. When the tumor size in any mouse reached the criteria for euthanasia, the mice were euthanized. V = 1/2 × L × S^2^, where L is the long diameter and S is the short diameter. The subcutaneous tumor tissues were isolated, photographed, and then fixed with OCT and formaldehyde. These tissues were subsequently subjected to Oil Red O staining, IHC, and H&E staining. For the footpad‐popliteal lymph node metastasis model, a total of 1 × 10^6^ cells were resuspended in 30 µL of PBS and injected into the foot pads of the mice. The mice were observed every three days. One month after injection, the mice were euthanized, and the popliteal lymph nodes on the side of the tumor were isolated, photographed, and fixed in formaldehyde. These tissues were then subjected to IHC and H&E staining.

### Statistical Analysis

GraphPad Prism (version 9) was utilized for data analysis. The values were presented as the means ± SDs. The data from the two unpaired groups were compared via a two‐tailed unpaired Student's *t*‐test. Statistical comparisons among three or more data groups were carried out with one‐way ANOVA followed by Dunnett's multiple comparison test, and survival analysis was performed via the log‐rank test. *p* < 0.05 was considered to indicate statistical significance.

### Ethics Statement

For human samples, this study was performed in accordance with the Declaration of Helsinki and was approved by the Medical Ethics Committee of Zhongnan Hospital of Wuhan University (approval number 2022177K). The animal study was approved by the Experimental Animal Welfare Ethics Committee at Zhongnan Hospital of Wuhan University (approval number ZN2023232).

## Conflict of Interest

The authors declare no conflict of interest.

## Author Contributions

K.X., S.C., H.X., and S.T. contributed equally to this work. K.X., S.C., H.X., Y.X., X.W., and G.W. designed the study. K.X., Y.X., and G.W. wrote the manuscript. K.X. and S.C. performed the cellular and biochemical experiments. K.X., S.C., H.X., H.W., and J.Y. performed the animal experiments. K.X., S.T., H.W., Y.W., M.L., J.Y., K.Q., and G.W. helped with data collection and assembly. K.X., S.T., L.J., Y.Z., Y.X., X.W., and G.W. performed data analysis and interpretation. All the authors corrected the final manuscript.

## Supporting information



Supporting Information

Supplemental Dataset 1

Supplemental Dataset 2

Supplemental Dataset 3

## Data Availability

The data that support the findings of this study are openly available in Gene Expression Omnibus (GEO) at https://www.ncbi.nlm.nih.gov/geo/query/acc.cgi?acc=GSE270143, reference number 270143.
