# Supplementary material for: RNF112 Facilitates Ubiquitin‐Mediated Degradation of c‐Myc, Suppressing Proliferation, Migration and Lipid Synthesis in Bladder Cancer
Source: Adv Sci (Weinh). 2025 Apr 3;12(20):2408311. doi: 10.1002/advs.202408311 (PMC12120744; doi:10.1002/advs.202408311)
Supplement: Supplementary file 1 — Supporting Information [file ADVS-12-2408311-s003.pdf]

ADVANCED SCIENCE
Open Access

Supporting Information

for *Adv. Sci.*, DOI 10.1002/advs.202408311

RNF112 Facilitates Ubiquitin-Mediated Degradation of c-Myc, Suppressing Proliferation, Migration and Lipid Synthesis in Bladder Cancer

Kangping Xiong, Siming Chen, Huimin Xu, Sheng Tu, Hong Weng, Yejinpeng Wang, Mingxing Li, Jingtian Yu, Kaiyu Qian, Lingao Ju, Yi Zhang, Yu Xiao, Xinghuan Wang* and Gang Wang**

Supplementary Information

RNF112 Facilitates Ubiquitin-Mediated Degradation of c-Myc, Suppressing Proliferation, Migration and Lipid Synthesis in Bladder Cancer

Figures S1-S11: Pages 2-21

Tables S1-S6: Pages 22-28

Description of Datasets S1-S3: Pages 29

Figures S1-S11

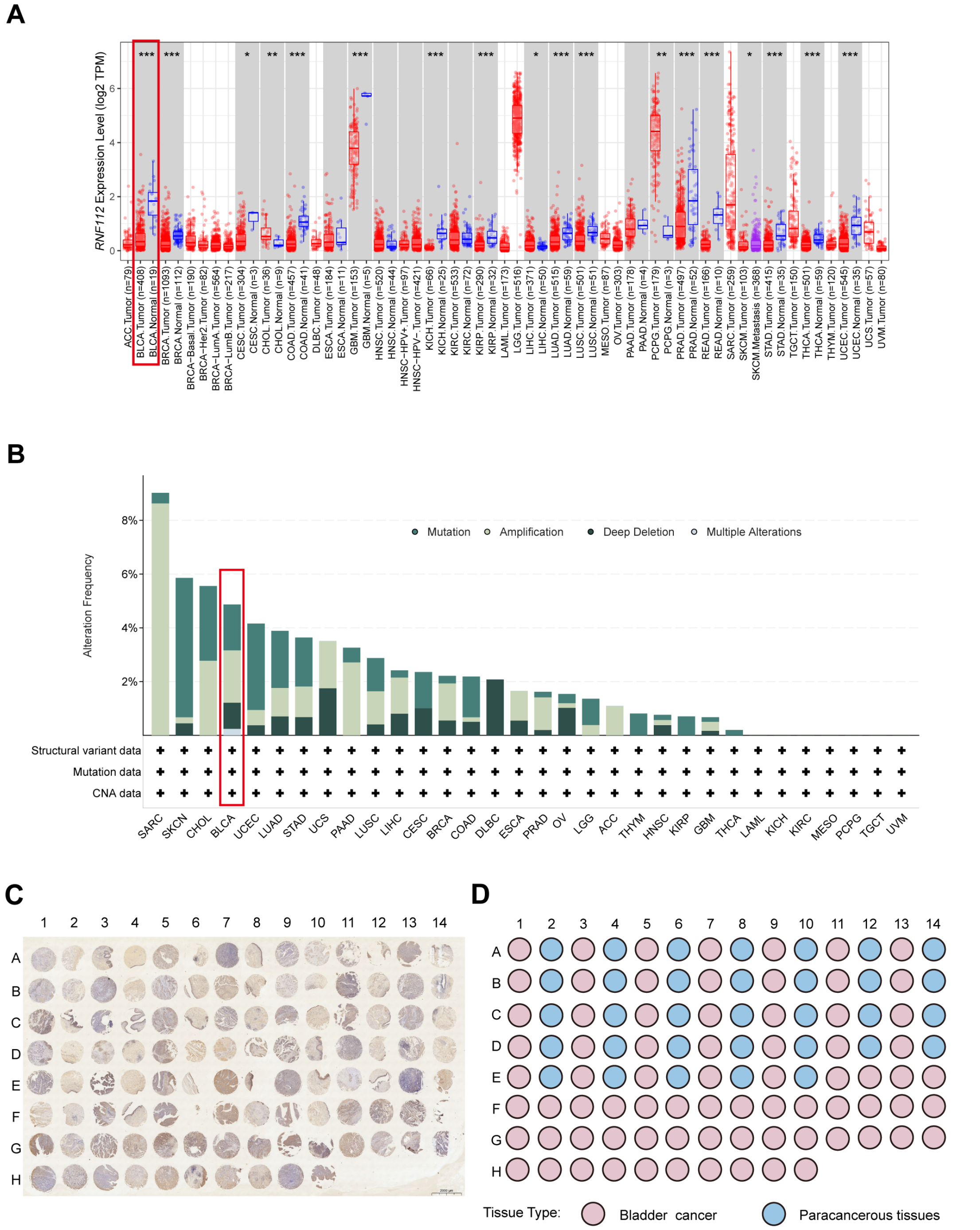

Figure S1. RNF112 is downregulated and mutated across cancers. Related to Figure 1.

(A) *RNF112* mRNA expression across cancers was analyzed with the TIMER 2.0 online database (*http://timer.comp-genomics.org/timer/*). **(B)** Mutations and deep deletions of

RNF112 across cancers were analyzed via the cBioPortal website (*https://www.cbioportal.org/*). **(C)** IHC images of HBlaU108Su01 cohort. Scale bar = 2 mm. **(D)** Distribution of BLCA and paracancerous tissues samples.

Abbreviations in the Figure S1: BLCA: bladder urothelial carcinoma; SARC: sarcoma; SKCN: skin cutaneous melanoma; CHOL: cholangiocarcinoma; UCEC: uterine corpus endometrial carcinoma; LUAD: lung adenocarcinoma; STAD: stomach adenocarcinoma; HNSC: head and neck squamous cell carcinoma; KIRC: kidney renal clear cell carcinoma; UCS: uterine carcinosarcoma; PAAD: pancreatic adenocarcinoma; LIHC: liver hepatocellular carcinoma; CESC: cervical squamous cell carcinoma; BRCA: breast invasive carcinoma; COAD: colorectal adenocarcinoma; DLBC: diffuse large B-cell lymphoma; PCPG: pheochromocytoma and paraganglioma; ESCA: esophageal adenocarcinoma; PRAD: prostate adenocarcinoma; OV: ovarian serous cystadenocarcinoma; LUSC: lung squamous cell carcinoma; ACC: adrenocortical carcinoma; THYM: thymoma; KIRP: kidney renal papillary cell carcinoma; GBM: glioblastoma multiforme; THCA: thyroid carcinoma; LAML: acute myeloid leukemia; KICH: kidney chromophobe; LGG: brain lower grade glioma; MESO: mesothelioma; TGCT: testicular germ cell tumors; UVM: uveal melanoma. *$p < 0.05$, **$p < 0.01$, ***$p < 0.001$.

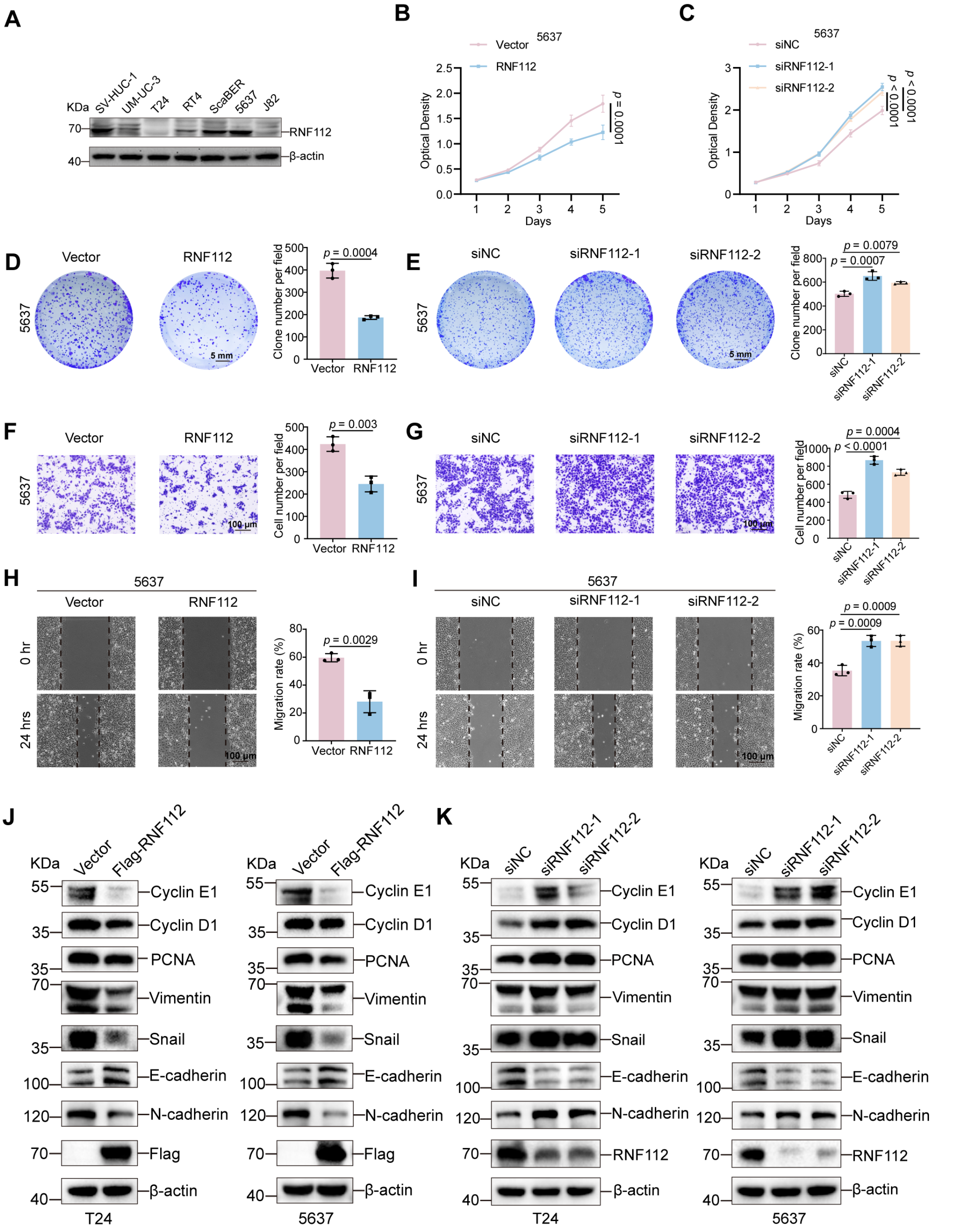

Figure S2. RNF112 inhibits BLCA growth and metastasis *in vitro*. Related to Figure 2.

(A) RNF112 protein expression in SV-HUC-1 cells and various BLCA cell lines was detected by Western blot analysis. **(B-C)** The viability of 5637 cells with RNF112 knockdown (B) or overexpression (C) was determined via CCK-8 assays (n = 6). **(D-E)** Representative images of plate colony formation assays after the overexpression (D)

or knockdown (E) of RNF112 in 5637 cells and the corresponding statistical graphs (n = 3). Scale bar = 5 mm. **(F-G)** Representative images of transwell migration assays after overexpression (F) or knockdown (G) of RNF112 in 5637 cells and the corresponding statistical graphs (n = 3). Scale bar = 100 μm. **(H-I)** Representative images of wound healing assays after the overexpression (H) or knockdown (I) of RNF112 in 5637 cells and the corresponding statistical graphs (n = 3). Scale bar = 100 μm. **(J-K)** Western blot analysis was performed to detect changes in proliferation-associated proteins and EMT-associated proteins in 5637 and T24 cells after overexpression (J) or knockdown (K) of RNF112. *p*-values were determined by two-tailed unpaired Student's t-test (B, D, F, and H) and one-way ANOVA with Dunnett's multiple comparisons (C, E, G, and I). The data are shown as the means ± SDs.

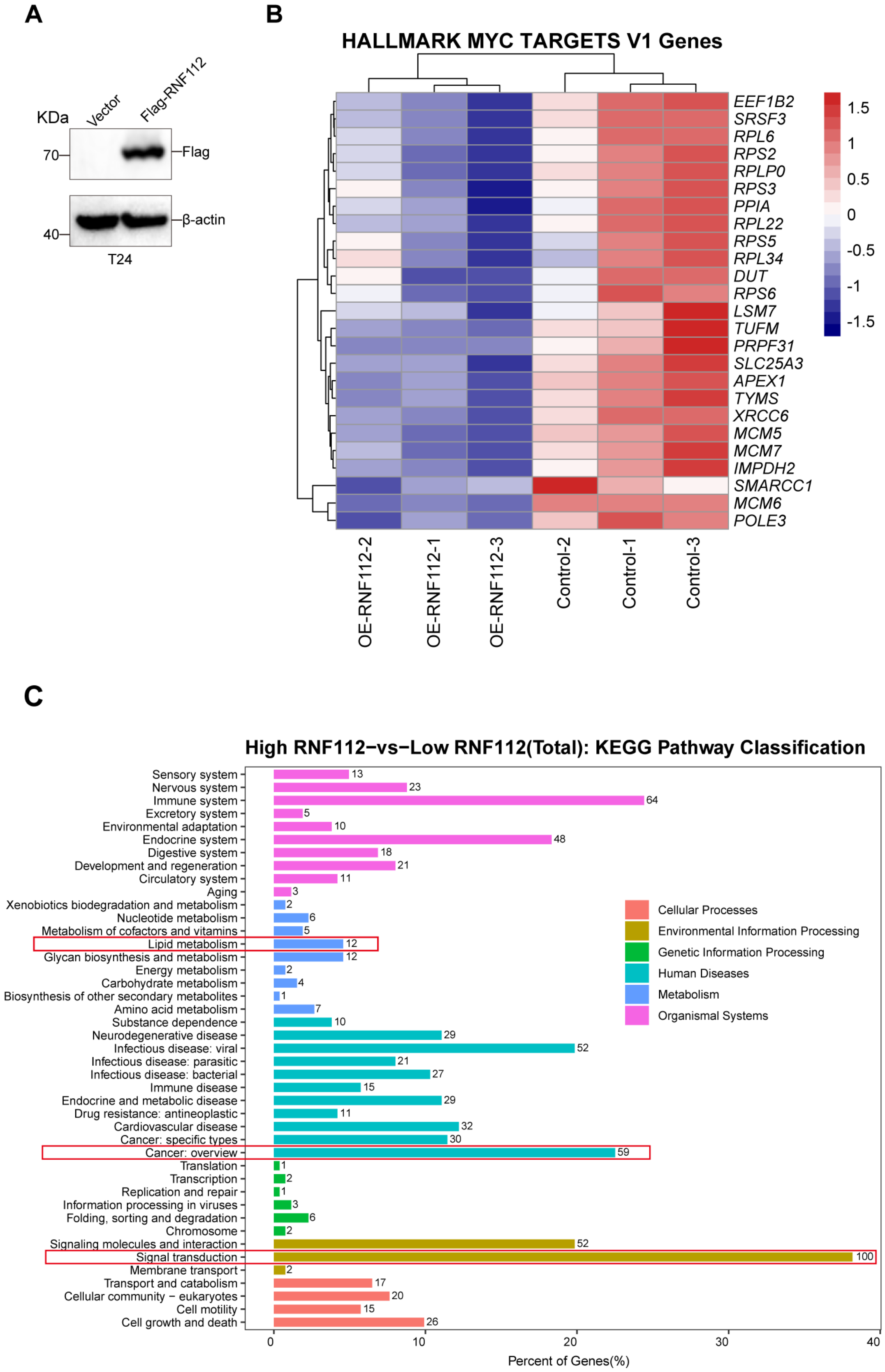

Figure S3. RNA-seq analysis revealed that RNF112 was associated with the MYC pathway and lipid synthesis in BLCA cells. Related to Figure 2 and Figure 3.

(A) Western blot analysis was used to verify the successful establishment of two stable T24-overexpressing cell lines (control and Flag-RNF112). **(B)** Heatmap diagram showing the top 25 genes in the HALLMARK MYC TARGETS V1 gene rankings in the control and RNF112-overexpressing groups from RNA-seq assays. p-value < 0.05 [or FDR < 0.25] was set as the threshold for the enrichment and reliability of the gene set. **(C)** KEGG pathway classification analysis of differentially expressed genes in the RNA-seq results.

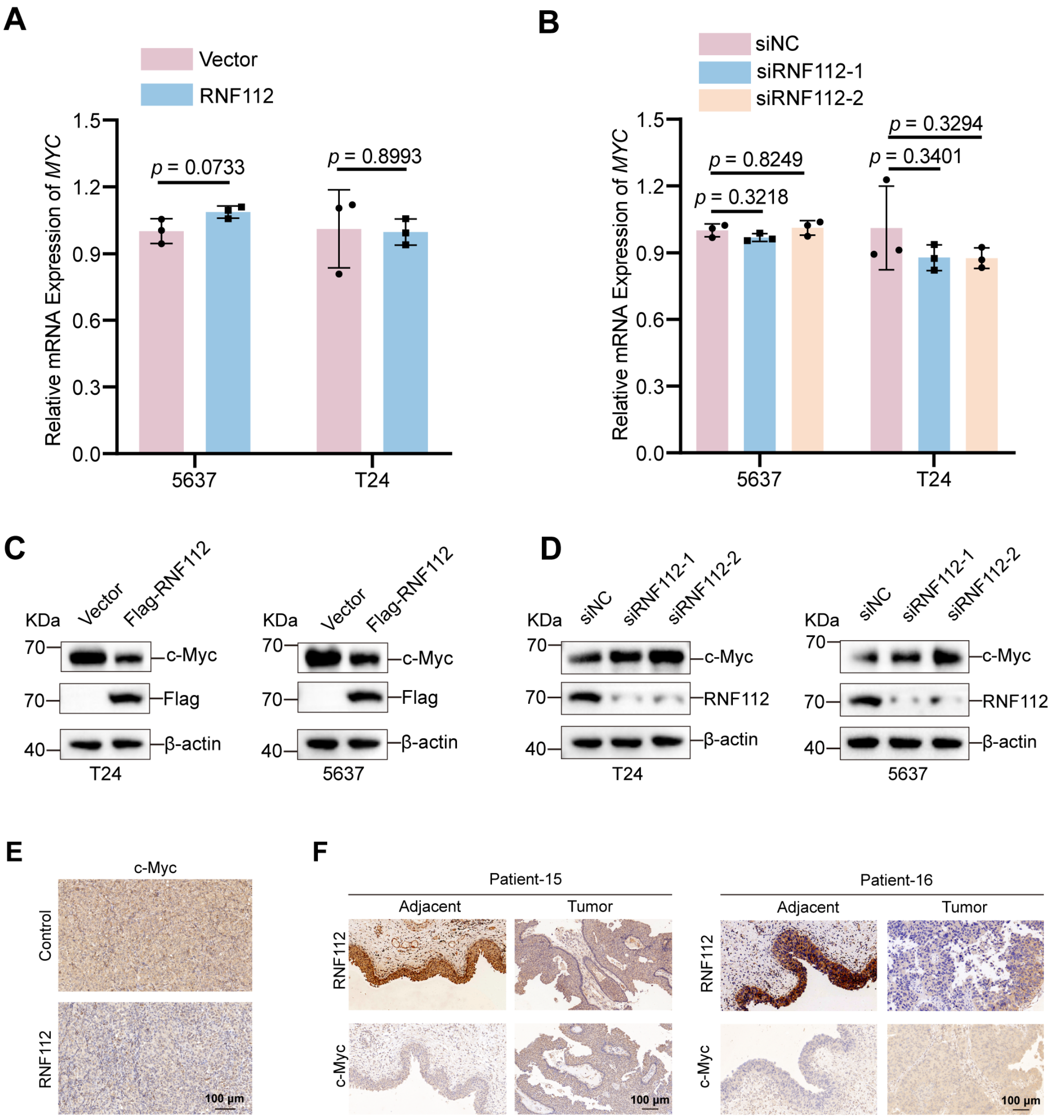

Figure S4. RNF112 downregulated c-Myc protein levels without affecting *MYC* mRNA levels. Related to Figure 4.

(A-B) Effects on *MYC* mRNA expression after the overexpression (A) or knockdown (B) of RNF112 in 5637 and T24 cells were detected via qRT-PCR (n = 3). **(C-D)** The effects of RNF112 overexpression (C) or knockdown (D) on the c-Myc protein in 5637 and T24 cells were detected by Western blot analysis. **(E)** Representative images of IHC staining of tumor tissue from subcutaneous xenografts in the control and RNF112-overexpressing groups. Scale bar = 100 μm. **(F)** Representative IHC staining of RNF112 and c-Myc in serial tumor tissue sections (n = 3) from Zhongnan Hospital. Scale bar = 100 μm. The *p*-values were determined by two-tailed unpaired Student's t-

test (A) and one-way ANOVA followed by Dunnett's multiple comparisons (B). The data are shown as the means ± SDs.

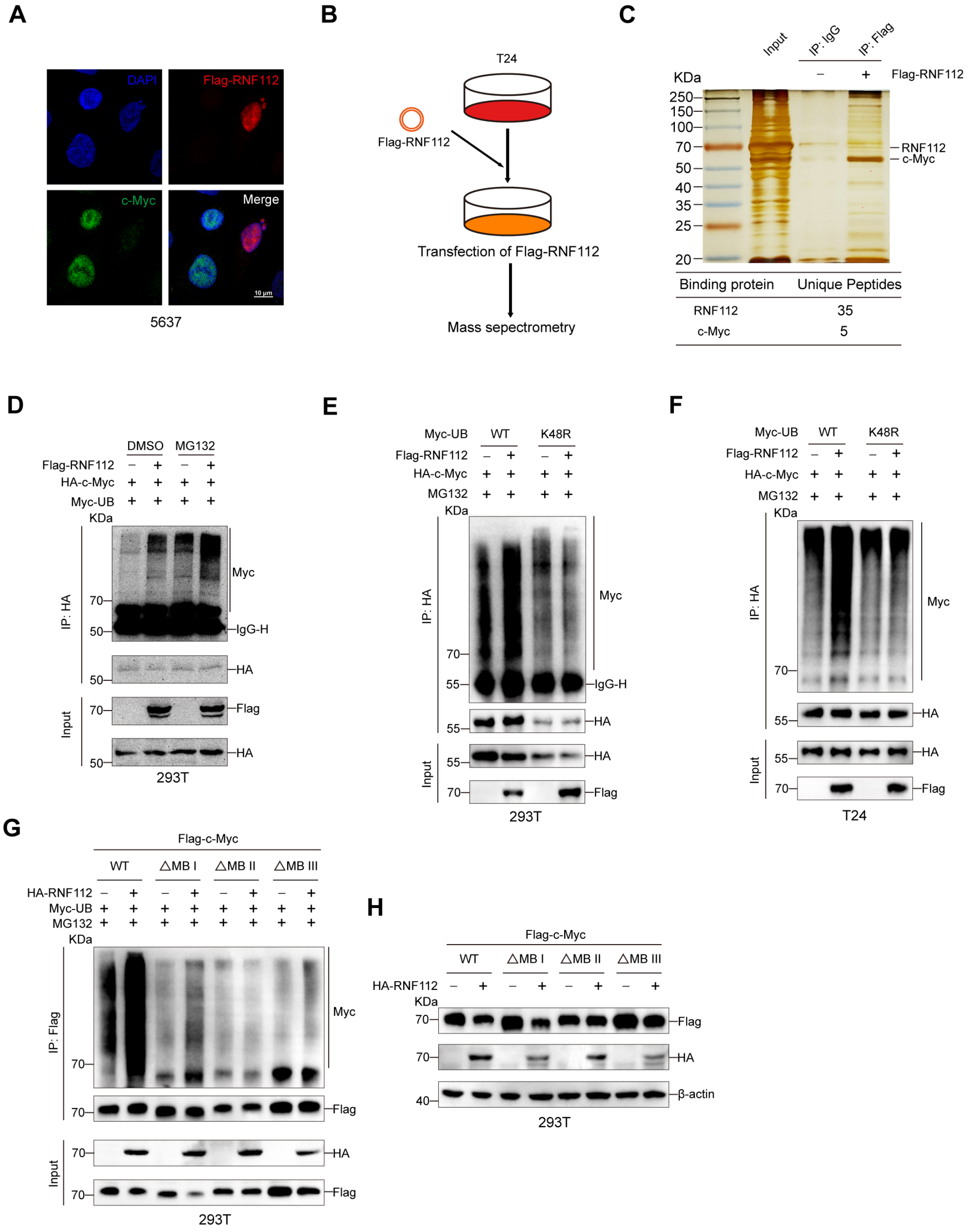

Figure S5. RNF112 bound to the MBII structural domain of c-Myc and promoted its degradation via ubiquitination. Related to Figure 5.

(A) Immunofluorescence staining was used to detect the expression and localization of Flag-RNF112 (red) and c-Myc (green) in 5637 cells, and the nuclei were stained with DAPI (blue). Scale bar = 10 μm. **(B)** Flow chart of the IP-MS analyses. **(C)** T24 cells were transfected with Flag-RNF112 plasmid and then subjected to IP experiments with

Flag antibody, followed by silver staining (top) and mass spectrometry (bottom). **(D)** The respective plasmids were transfected into 293T cells for 48 hrs, DMSO or MG132 (10 μM) was added for 8 hrs, and a ubiquitination assay was used to determine the effect of RNF112 on c-Myc ubiquitination. **(E-F)** The indicated plasmids were transfected into 293T (E) and T24 (F) cells for 48 hrs, followed by the addition of MG132 (10 μM) for 8 hrs. Ubiquitination assays were performed to detect the polyubiquitination of the c-Myc protein. **(G)** The respective plasmids were transfected into 293T cells for 48 hrs, and MG132 (10 μM) was added for 8 hrs. Anti-Flag antibody was added to perform the ubiquitination assays. **(H)** The indicated plasmids were transfected into 293T cells for 48 hrs. Changes in the level of the Flag-c-Myc protein in each group of cells were detected by Western blot.

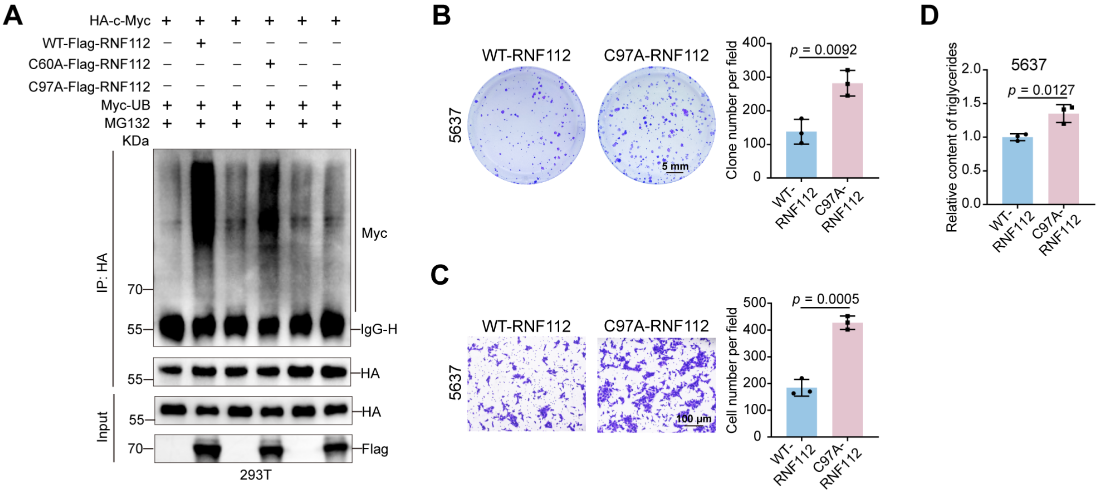

Figure S6. Functional effects of WT-RNF112 and C97A-RNF112 overexpression in 5637 cells.

(A) 293T cells were transfected with the respective plasmids for 48 hrs, and MG132 (10 μM) was added for 8 hrs. An anti-HA antibody was added to perform the ubiquitination assays. **(B)** Representative images of plate colony formation assays after the overexpression of WT-RNF112 or C97A-RNF112 and the corresponding statistical graphs (n = 3) in 5637 cells. Scale bar = 5 mm. **(C)** Representative images of transwell migration assays after overexpression of WT-RNF112 or C97A-RNF112 and the corresponding statistical graphs (n = 3) in 5637 cells. Scale bar = 100 μm. **(D)** Statistical graphs showing the relative triglyceride content after the overexpression of WT-RNF112 or C97A-RNF112 in 5637 cells. *p*-values were determined by two-tailed unpaired Student's t-test (B, C, and D). The data are shown as the means ± SDs.

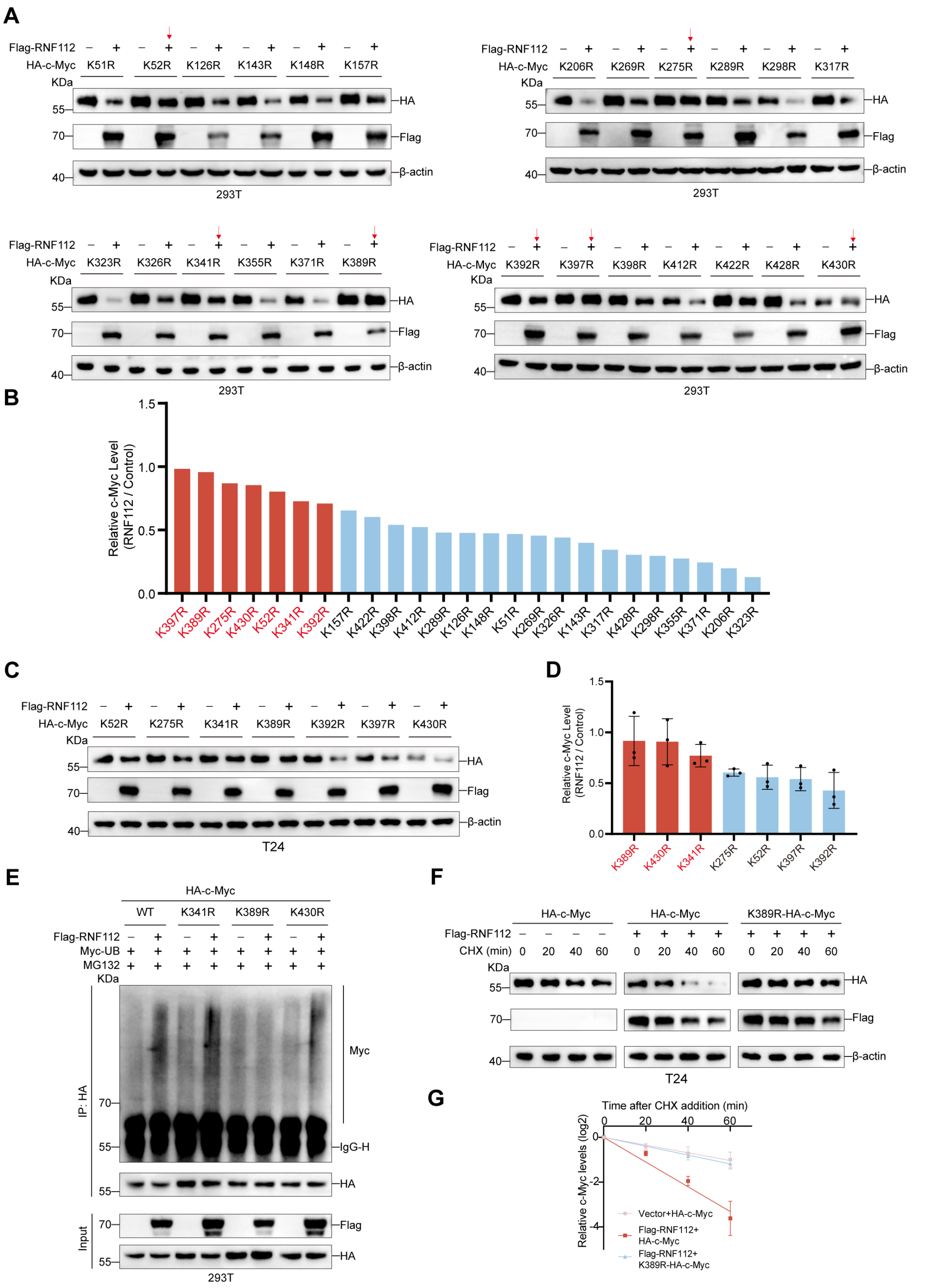

Figure S7. RNF112 promotes the ubiquitination of c-Myc at C389 Related to Figure 5.

(A) The respective plasmids were transfected into 293T cells for 48 hrs, and Western blot analysis was performed to detect the expression of various exogenous HA-c-Myc mutants. **(B)** Relative c-Myc protein expression levels in each group shown in Figure

S6A. **(C)** The respective plasmids were transfected into T24 cells for 48 hrs. Western blot analysis was used to detect the protein expression of HA-c-Myc in the K52R, K275R, K341R, K389R, K392R, K397R, and K430R groups. **(D)** Relative c-Myc protein expression levels in each group shown in Figure S6C. **(E)** The respective plasmids were transfected into 293T cells for 48 hrs. MG132 (10 μM) was added for 8 hrs. An anti-HA antibody was added to perform the ubiquitination assays. **(F)** Western blot analysis was conducted by transfecting the respective plasmids into T24 cells for 48 hrs and incubating them with CHX (50 μg/mL) for the indicated times (n = 3). **(G)** Statistics of the relative grayscale values of the c-Myc protein shown in Figure S7F.

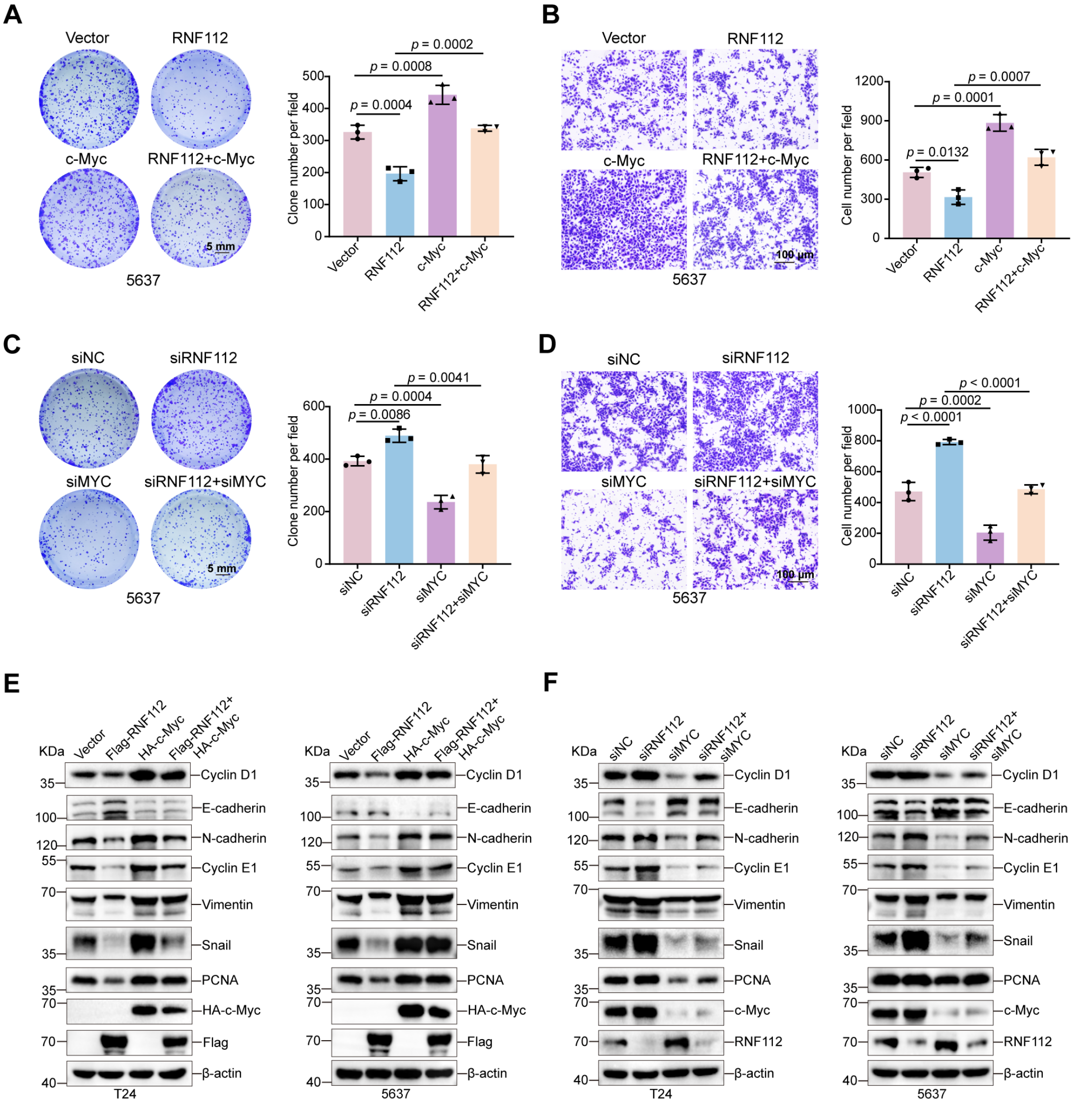

Figure S8. RNF112 inhibits BLCA progression via c-Myc *in vitro*. Related to Figure 6.

(A) Representative images (left panel) and statistical analysis (right panel) of colony formation assays in 5637 cells overexpressing RNF112 and c-Myc, respectively, or in combination (n = 3). Scale bar = 5 mm. **(B)** Representative images (left panel) and statistical analysis (right panel) of transwell assays in 5637 cells overexpressing RNF112 and c-Myc, respectively, or in combination (n = 3). Scale bar = 100 μm. **(C)** Representative images (left panel) and statistical analysis (right panel) of colony formation assays in 5637 cells with RNF112 and c-Myc knockdown separately or in combination (n = 3). Scale bar = 5 mm. **(D)** Representative images (left panel) and statistical analysis (right panel) of transwell assays of 5637 cells with RNF112 and c-

Myc knockdown separately or in combination (n = 3). Scale bar = 100 µm. **(E-F)** Western blot analysis was performed to detect changes in the expression of proliferation-associated proteins and EMT-associated proteins in BLCA cells with RNF112 and c-Myc overexpression (E) or RNF112 and c-Myc knockdown (F). The *p*-values were determined by one-way ANOVA followed by Dunnett's multiple comparisons test (A, B, C, and D). The data are shown as the means ± SDs.

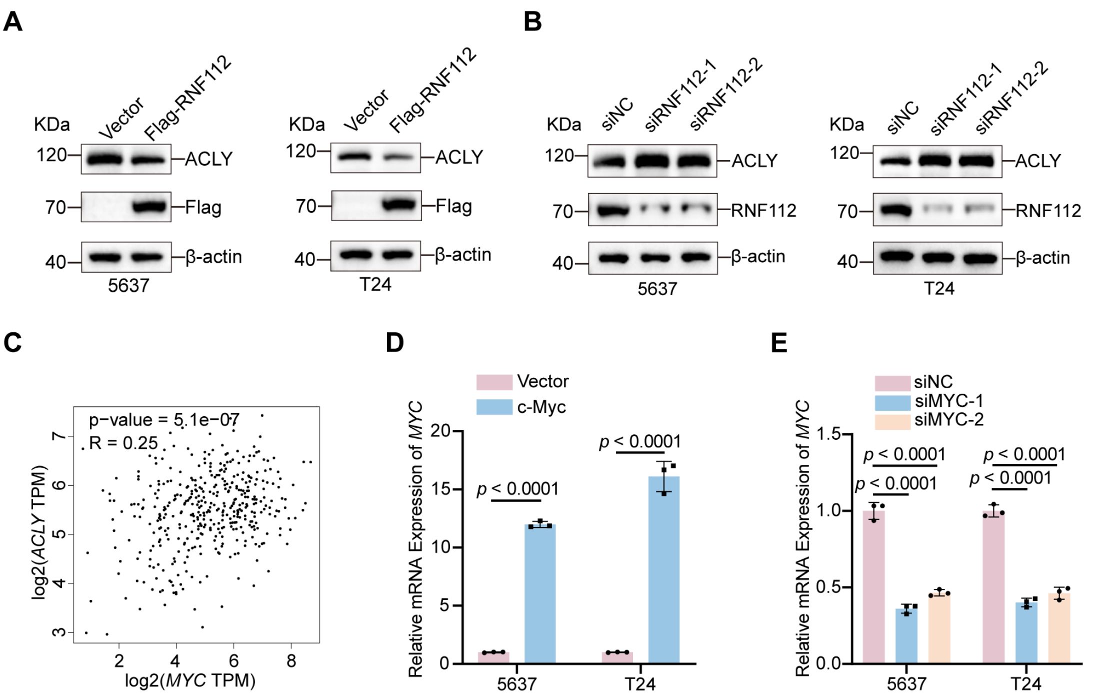

Figure S9. RNF112 was negatively correlated with ACLY protein levels, and *MYC* mRNA was positively correlated with *ACLY* mRNA. Related to Figure 7.

(A-B) Effects of RNF112 overexpression (A) or knockdown (B) on the ACLY protein detected in BLCA cells by Western blot. **(C)** Spearman's correlation analysis was used to estimate the correlation between *ACLY* mRNA and *MYC* mRNA in BLCA tissues in the GEPIA dataset. **(D-E)** qRT-PCR was used to validate the efficiency of MYC overexpression (D) or knockdown (E) in the 5637 and T24 cell lines (n = 3). *p*-values were determined by two-tailed unpaired Student's t-test (D) and one-way ANOVA followed by Dunnett's multiple comparisons (E). The data are shown as the means ± SDs.

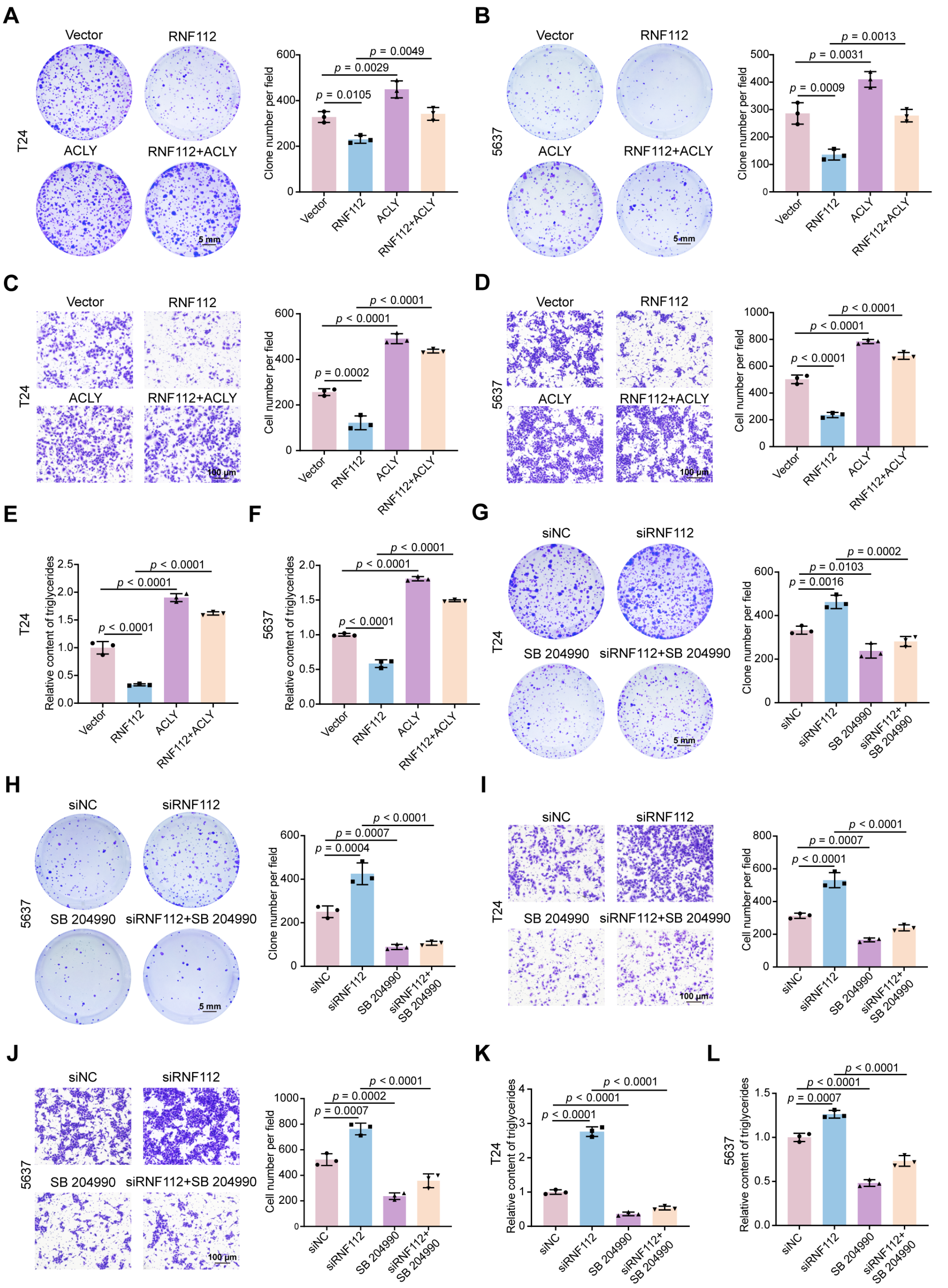

Figure S10. RNF112 inhibits proliferation, metastasis, and lipid synthesis in BLCA via ACLY.

(A-B) Representative images and statistical analysis of colony formation assays in T24 cells (A) and 5637 cells (B) overexpressing RNF112 and ACLY (n = 3). Scale bar = 5 mm. **(C-D)** Representative images and statistical analysis of transwell migration assays

in T24 cells (C) and 5637 cells (D) overexpressing RNF112 and ACLY (n = 3). Scale bar = 100 µm. **(E-F)** Statistical graphs showing the relative triglyceride levels in T24 cells (E) and 5637 cells (F) overexpressing RNF112 and ACLY. **(G-H)** Representative images and statistical analysis of colony formation assays in T24 cells (G) and 5637 cells (H) with RNF112 knockdown combined with SB 204990 (30 µM) treatment (n = 3). Scale bar = 5 mm. **(I-J)** Representative images and statistical analysis of Transwell assays in T24 cells (I) and 5637 cells (J) with RNF112 knockdown combined with SB 204990 (30 µM) treatment (n = 3). Scale bar = 100 µm. **(K-L)** Statistical graphs showing the relative triglyceride levels in T24 cells (K) and 5637 cells (L) in which RNF112 was knocked down in combination with SB 204990 (30 µM) treatment (n = 3). *p*-values were determined by one-way ANOVA followed by Dunnett's multiple comparisons test (A-L). The data are shown as the means ± SDs.

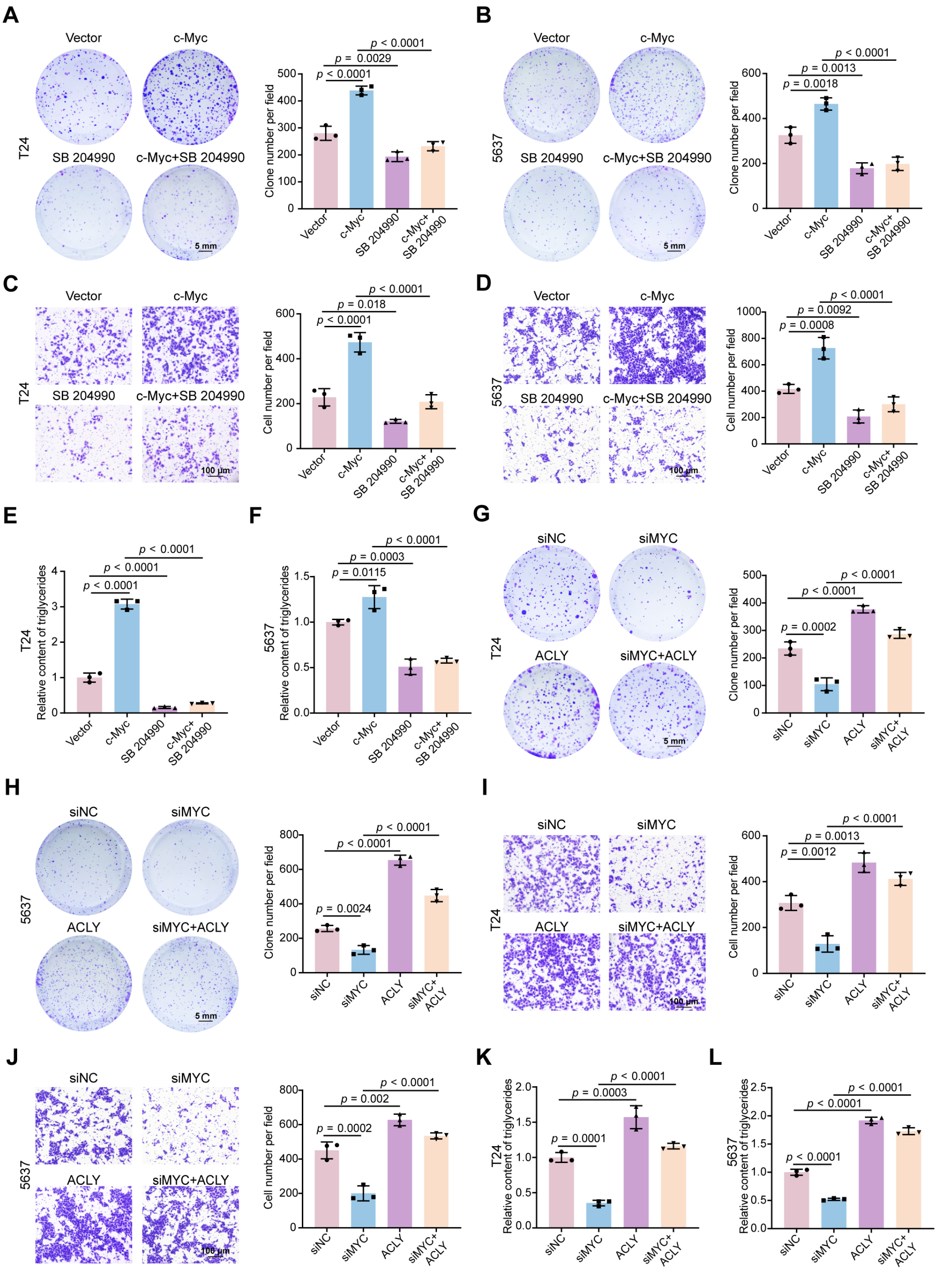

Figure S11. c-Myc promotes proliferation, metastasis, and lipid synthesis in BLCA via ACLY.

(A-B) Representative images and statistical analysis of colony formation assays in T24 cells (A) and 5637 cells (B) overexpressing c-Myc combined with SB 204990 (30 μM) treatment (n = 3). Scale bar = 5 mm. **(C-D)** Representative images and statistical

analysis of transwell migration assays in T24 cells (C) and 5637 cells (D) overexpressing c-Myc combined with SB 204990 (30 μM) treatment (n = 3). Scale bar = 100 μm. **(E-F)** Statistical graphs showing the relative triglyceride levels in T24 cells (E) and 5637 cells (F) overexpressing c-Myc combined with SB 204990 (30 μM) treatment (n = 3). **(G-H)** Representative images and statistical analysis of colony formation assays in T24 cells (G) and 5637 cells (H) with c-Myc knockdown combined with ACLY overexpression (n = 3). Scale bar = 5 mm. **(I-J)** Representative images and statistical analysis of Transwell assays in T24 cells (I) and 5637 cells (J) with c-Myc knockdown combined with ACLY overexpression (n = 3). Scale bar = 100 μm. **(K-L)** Statistical graphs showing the relative triglyceride levels in T24 cells (K) and 5637 cells (L) in which c-Myc was knocked down and ACLY was overexpressed. *p*-values were determined by one-way ANOVA followed by Dunnett's multiple comparisons test (A-L). The data are shown as the means ± SDs.

Tables S1-S6

Table S1. Clinicopathological characteristics of the HBlaU108Su01 cohort.

	Variables	Total (n = 68)	RNF112 high expression (n = 34)	RNF112 low expression (n = 34)	*p*-value	Statistics method
Gender (%)	female	10 (14.70)	3 (8.82)	7 (20.59)	0.3047	Fisher's exact
	male	58 (85.30)	31 (91.18)	27 (79.41)		
Age (year) (%)	≤ 65	25 (37.31)	14 (41.18)	11 (33.33)	0.5069	Chi-square
	> 65	42 (62.69)	20 (58.82)	22 (66.67)		
Subtype (%)	NMIBC	17 (26.98)	13 (39.39)	4 (13.33)	**0.0250**	Fisher's exact
	MIBC	46 (73.02)	20 (60.61)	26 (86.67)		
AJCC stage (%)	< Stage II	11 (18.64)	8 (26.67)	3 (10.34)	0.1806	Fisher's exact
	≥ Stage II	48 (81.36)	22 (73.33)	26 (89.66)		
T (%)	< T2	17 (26.98)	13 (39.39)	4 (13.33)	**0.0250**	Fisher's exact
	≥ T2	46 (73.02)	20 (60.61)	26 (86.67)		
N (%)	N0	47 (81.03)	23 (79.67)	24 (85.71)	0.5079	Fisher's exact
	N ≥ 1	11 (18.97)	7 (23.33)	4 (14.29)		
Tumor Size (%)	≤ 3 cm	21 (34.43)	12 (37.50)	9 (31.03)	0.5956	Chi-square
	> 3 cm	40 (65.57)	20 (62.50)	20 (68.97)		

RNF112 expression group: The median RNF112 histochemistry score was the cutoff value.
Tumor size: longest diameter, cm.
Statistical significance: Determined by two-tailed chi-square tests or two-tailed Fisher's exact tests. No adjustments were made for multiple comparisons.

Table S2. Clinicopathological characteristics of the Zhongnan Hospital cohort.

	Patients	Age	Gender	Subtype	Grade	TNM stage
qRT-PCR	Patient-1	83	Male	MIBC	High	T3N0Mx
	Patient-2	66	Male	MIBC	High	T2bN0Mx
	Patient-3	76	Female	MIBC	High	T2bN0Mx
	Patient-4	64	Male	MIBC	High	T2aN0Mx
	Patient-5	80	Male	NMIBC	High	T1N0Mx
	Patient-6	71	Male	MIBC	High	T4aN1M1
	Patient-7	55	Male	MIBC	High	T3N0Mx
	Patient-8	69	Male	MIBC	High	T4N0Mx
	Patient-9	59	Male	MIBC	High	T4aN0Mx
	Patient-10	69	Male	NMIBC	Low	T1N0Mx
	Patient-11	61	Male	NMIBC	High	T1N0Mx
	Patient-12	73	Male	MIBC	High	T3N0Mx
	Patient-13	65	Male	MIBC	High	T2bN0Mx
	Patient-14	51	Male	MIBC	High	T3bN0Mx
	Patient-15	69	Male	MIBC	High	T4N0Mx
	Patient-16	68	Male	MIBC	High	T2aN0Mx
	Patient-17	68	Male	MIBC	High	T2bN1Mx
	Patient-18	79	Male	NMIBC	Low	T1N0Mx
	Patient-19	76	Female	MIBC	High	T2bN0Mx
	Patient-20	67	Male	MIBC	High	T3bN2Mx
	Patient-21	70	Male	MIBC	High	T2bN0Mx
	Patient-22	75	Male	MIBC	High	T2bNxMx
	Patient-23	87	Male	MIBC	High	T2N0Mx
	Patient-24	80	Male	MIBC	High	T2N0MX
	Patient-25	69	Male	NMIBC	High	T1N0Mx
	Patient-26	84	Male	MIBC	High	T3aN0Mx
	Patient-27	65	Male	MIBC	High	T4N0Mx
	Patient-28	63	Female	NMIBC	High	T1N0Mx
	Patient-29	57	Male	NMIBC	High	T1N0Mx
	Patient-30	71	Male	MIBC	High	T3aN1Mx
	Patient-31	64	Male	MIBC	High	T2N0Mx
	Patient-32	65	Male	NMIBC	High	TisN0Mx
	Patient-33	73	Male	MIBC	High	T3N0Mx
	Patient-34	69	Male	NMIBC	High	T1N0Mx
	Patient-35	74	Male	MIBC	High	T3N0Mx
	Patient-36	47	Female	NMIBC	High	T1N0Mx

	Patient-37	73	Male	MIBC	High	T2N0Mx
	Patient-38	50	Male	NMIBC	High	T1N0Mx
	Patient-39	82	Male	NMIBC	High	T1N0Mx
	Patient-40	55	Female	NMIBC	High	T1N0Mx
	Patient-41	78	Male	MIBC	High	T4N1Mx
	Patient-42	67	Female	NMIBC	Low	T1N0Mx
	Patient-43	93	Male	MIBC	High	T4aNxMx
	Patient-44	84	Male	MIBC	High	T3aN0Mx
	Patient-45	75	Male	NMIBC	High	T1aNxMx
	Patient-46	59	Male	MIBC	High	T2aN0Mx
	Patient-47	70	Male	NMIBC	High	T1N0Mx
	Patient-48	82	Female	MIBC	High	T3N1Mx
	Patient-49	55	Male	NMIBC	High	T1aN0Mx
	Patient-50	72	Male	MIBC	High	T4aN0Mx
IHC	Patient-51	66	Male	NMIBC	High	T1N0Mx
	Patient-52	66	Male	NMIBC	Low	T1N0Mx
	Patient-53	60	Male	NMIBC	Low	T1N0Mx

qRT-PCR: quantitative reverse transcription PCR.
IHC: immunohistochemistry.

Table S3. The siRNA sequences used in this study.

Gene	siRNA No.	siRNA sequence (5'-3')
RNF112	si#1	CCUGAGUGCCGGAAGAUAUTT
	si#2	CCUUCCUCCUCAACCAUUUTT
MYC	si#1	GCUUGUACCUGCAGGAUCUTT
	si#2	GGAAGAAAUCGAUGUUGUUTT

Table S4. Primers used for the qRT-PCR assay.

Gene	Forward primer (5’-3’)	Reverse primer (5’-3’)
RNF112	GCTTGGCAAACGGGAGAGAA	CAGGCAGATGGAGCAGGTAG
β-actin	CATGTACGTTGCTATCCAGGC	CTCCTTAATGTCACGCACGAT
MYC	GTCAAGAGGCGAACACACAAC	TTGGACGGACAGGATGTATGC
ACLY	GAAGCTGACCTTGCTGAACC	CTGCCTCCAATGATGAGGAT
ACACA	TCACACCTGAAGACCTTAAAGCC	AGCCCACACTGCTTGTACTG
SCD	TCATAATTCCCGACGTGGCT	CCCAGAAATACCAGGGCACA
HMGCR	TGGCAACAACAGAAGGTTGTC	CGTGCAAATCTGCTAGTGCT
FASN	ACAGCGGGGAATGGGTACT	GACTGGTACAACGAGCGGAT
HMGCS1	GATGTGGGAATTGTTGCCCTT	ATTGTCTCTGTTCCAACTTCCAG
CPT1A	TCCAGTTGGCTTATCGTGGTG	TCCAGAGTCCGATTGATTTTTGC
CPT1B	CCTGCTACATGGCAACTGCTA	AGAGGTGCCCAATGATGGGA
SREBF1	ACAGTGACTTCCCTGGCCTAT	GCATGGACGGGTACATCTTCAA

qRT-PCR: quantitative reverse transcription PCR.

Table S5. Details of the antibodies used in this study.

Protein	Catalog No.	Source	Dilution or amount
GFP	SC-9996	Santa Cruz	IF/1:200
Flag	AE092	ABclonal	IP/1 μg WB/1:1000
HA	AE105	ABclonal	IP/1 μg WB/1:1000
Flag	F1804	Sigma	IP/1 μg WB/1:1000 IF/1:200
HA	TA180128	Origene	IP/1 μg WB/1:1000
Myc-tag	AE010	ABclonal	WB/1:1000
RNF112	A15333	ABclonal	IP/1 μg WB/1:1000 IHC/1:100
c-Myc	18583	Cell Signaling Technology	IP/1 μg WB/1:1000 IF/1:200
c-Myc	ab32072	Abcam	ChIP/8 μg WB/1:1000 IHC/1:100
Snail	3879S	Cell Signaling Technology	WB/1:1000
Cyclin D1	ab134175	Abcam	WB/1:1000
Cyclin E1	ab33911	Abcam	WB/1:1000
Vimentin	5741S	Cell Signaling Technology	WB/1:1000
Ki67	ab16667	Abcam	IHC/1:200
GST	10000-0-AP	Proteintech	WB/1:1000
His	66005-1-Ig	Proteintech	WB/1:5000
ACLY	A3719	ABclonal	WB/1:1000
E-cadherin	3195	Cell Signaling Technology	WB/1:500
N-cadherin	13116	Cell Signaling Technology	WB/1:500
PCNA	10205-2-AP	Proteintech	WB/1:1000
β-Actin	AC026	ABclonal	WB/1:1000

WB: Western blot.
IF: immunofluorescence.
IHC: immunohistochemistry.
ChIP: Chromatin immunoprecipitation.

Table S6. Primers used for the ChIP assay.

Amplicons	Forward primer (5'-3')	Reverse primer (5'-3')
ACLY-P1	GTCAGATGTGTAGTTTTGTGGTTAG	CCCAGTGCATACTCAATTTTATAGAT
ACLY-P2	AATAATGTTGCTGGTTGGTGCAGCA	TTGGGATTACAGGCATGTCCCACC
ACLY-P3	GCCGGTTAATCATGAAACATTTAAGT	TTCACTGATTTTTGATGGACACACT
ACLY-P4	GGGCCAGGGAGTGATGATTGCG	AACTCCAGAGAGAACCGCCAGA
LDHA	TCCTGACTCAGGCTCATGGC	AGACAACCGACCGGCAGA

ChIP: Chromatin immunoprecipitation.

Description of Datasets S1-S3

Dataset S1. Details of the hallmark pathways enriched by GSEA.

Pathways enrichment analysis of the gene expression data obtained from RNA-seq (GSE270143) was performed via hallmark gene sets.

Dataset S2. Differential metabolites.

Differential metabolites from LC-MS-based untargeted metabolomics analysis using BLCA T24 cells overexpressing RNF112 and control cells. Metabolites were considered significantly different if they met the criteria of p-value < 0.05 and a fold change (FC) ≥ 1.2 or ≤ 1/1.2.

Dataset S3. IP-MS identification of RNF112-associated proteins.

T24 cells were transfected with Flag-RNF112 plasmid and then subjected to IP experiments with Flag antibody, followed by mass spectrometry.
